# A neural network model for online one-shot storage of pattern sequences

**DOI:** 10.1371/journal.pone.0304076

**Published:** 2024-06-20

**Authors:** Jan Melchior, Aya Altamimi, Mehdi Bayati, Sen Cheng, Laurenz Wiskott

**Affiliations:** Institute for Neural Computation, Faculty of Computer Science, Ruhr University Bochum, Bochum, Germany; Plymouth University, UNITED KINGDOM

## Abstract

Based on the CRISP theory (Content Representation, Intrinsic Sequences, and Pattern completion), we present a computational model of the hippocampus that allows for online one-shot storage of pattern sequences without the need for a consolidation process. In our model, CA3 provides a pre-trained sequence that is hetero-associated with the input sequence, rather than storing a sequence in CA3. That is, plasticity on a short timescale only occurs in the incoming and outgoing connections of CA3, not in its recurrent connections. We use a single learning rule named Hebbian descent to train all plastic synapses in the network. A forgetting mechanism in the learning rule allows the network to continuously store new patterns while forgetting those stored earlier. We find that a single cue pattern can reliably trigger the retrieval of sequences, even when cues are noisy or missing information. Furthermore, pattern separation in subregion DG is necessary when sequences contain correlated patterns. Besides artificially generated input sequences, the model works with sequences of handwritten digits and natural images. Notably, our model is capable of improving itself without external input, in a process that can be referred to as ‘replay’ or ‘offline-learning’, which helps in improving the associations and consolidating the learned patterns.

## 1 Introduction

Since the discovery of place cells in the rodent hippocampal formation, the hippocampus has become one of the most extensively studied regions in the brain. Unlike in other brain regions, the response of neurons in the hippocampal formation of rodents shows a direct correlation to the temporo-spatial location of the rodent in the environment, which makes them an excellent object of study for how rodents orient and navigate [1]. On the other hand, the hippocampus is also involved in forming new episodic memories [2], making it an excellent object of study for memory formation in the human brain [2–4]. A prominent example is patient H.M. (Henry Gustav Molaison), whose hippocampus was almost completely removed in an attempt to cure his epileptic seizures. After this surgery, he could not form new episodic memories anymore [5, 6].

The anatomical structure of the hippocampal formation can be divided roughly into three major subregions: Dentate Gyrus (DG), Cornu Ammonis 1 (CA1), and Cornu Ammonis 3 (CA3), which are connected in a feed-forward manner [7]. The hippocampus receives input from and sends output back to the Entorhinal Cortex (EC), which itself receives input from and sends output back to many brain regions. DG contains a large number of granule cells [8], whereas CA1 and CA3 contain pyramidal cells, and the latter is well known for its recurrent collaterals. For this reason, CA3 has been suggested to function as an auto-associative memory network that stores patterns via plasticity in CA3 and can retrieve memories based on incomplete or corrupted cues [9–13]. Since DG granule cells are sparsely active, it has been suggested to perform pattern separation to reduce the overlap between stored patterns in CA3 [9–12]. This set of assumptions has become known as the standard framework [14] over the last decades. Some experiments have indeed provided evidence that CA3 is required for successful cue-based retrieval of memories [15]. However, experiments have also shown that the hippocampus is involved in learning patterns in temporal order similar to episodic memories [2, 16], which the standard framework does not naturally account for. The proposed CRISP (Content Representation, Intrinsic Sequences, and Pattern Completion) theory [16] explicitly accounts for sequence memory in the hippocampus. In this theory, neuronal sequences, representing episodic memories, are hetero-associated with intrinsic sequences in CA3. In contrast to the standard framework, CRISP does not claim that plasticity in CA3 is required during memory encoding. Notice that the idea of associating an input sequence with an intrinsic sequence has been proposed previously [17, 18]. Computational models based on CRISP have previously been shown to be capable of successfully storing and retrieving sequences of memories [16, 19]. Intrinsic sequences in CA3 also provide an explanation for the phenomenon of pre-play observed in the hippocampal formation, where sequences of place cell activation are observed before the animal ever experienced the nvironment [20, 21].

Undeniably, the brain must be able to instantaneously store new memories. Surprisingly, most computational studies on memory in the hippocampus ignore this online one-shot learning aspect, i.e. storing one pattern at a time through a single update. Instead, many models store patterns in an offline batch mode, i.e. all patterns are temporarily stored in an external buffer and later encoded in the network at the same time. This difference is highly significant since online storage in artificial neural networks is much more difficult than offline storage. Online storage has to avoid catastrophic forgetting [22] and at the same time allow for the integration of new information while forgetting old information. It is worth noting here that online learning implies seeing a single pattern at a time to update the network’s dynamics, but this pattern might be used again later, while one-shot means that the pattern is seen and used only once. While a few studies have addressed online storage of static patterns in auto-associative memories (e.g. [23]), storage of memory sequences has been studied almost exclusively in the offline mode (e.g. [19, 24]). The one-shot learning aspect of sequences has also been studied in the context of replay by [25] and dendrite processing by [26]. In this work, we explicitly address the two aspects at the same time, by proposing a simple hippocampal model for online one-shot storage of pattern sequences that is based on the CRISP theory. We assign the role of pattern separation to DG, which is not only consistent with the standard framework but also with the role of ‘context reset’ CRISP assigns to DG, however with subtle differences. Subregion CA1 is not yet included in the model and is postponed to future work.

We show that using the Hebbian descent learning rule [27] and the hetero-association with intrinsic sequences enables the model to successfully perform one-shot storage of pattern sequences that can be recalled from a single cue. Furthermore, the model shows a reasonable rate of forgetting, allowing it to continuously store new patterns without interference. We show decorrelation in DG is necessary for hetero-associating input sequences of correlated patterns and can be achieved in a very simple way. Our model has the notable property to improve itself based on the ‘replay’ process that does not require external input. Finally, we show that the required average activity in CA3 decreases with increasing model size so that large models function properly when using the average activity recorded from the rat hippocampus.

## 2 Model description

### 2.1 System-level view of the hippocampus model

**Fig 1:**
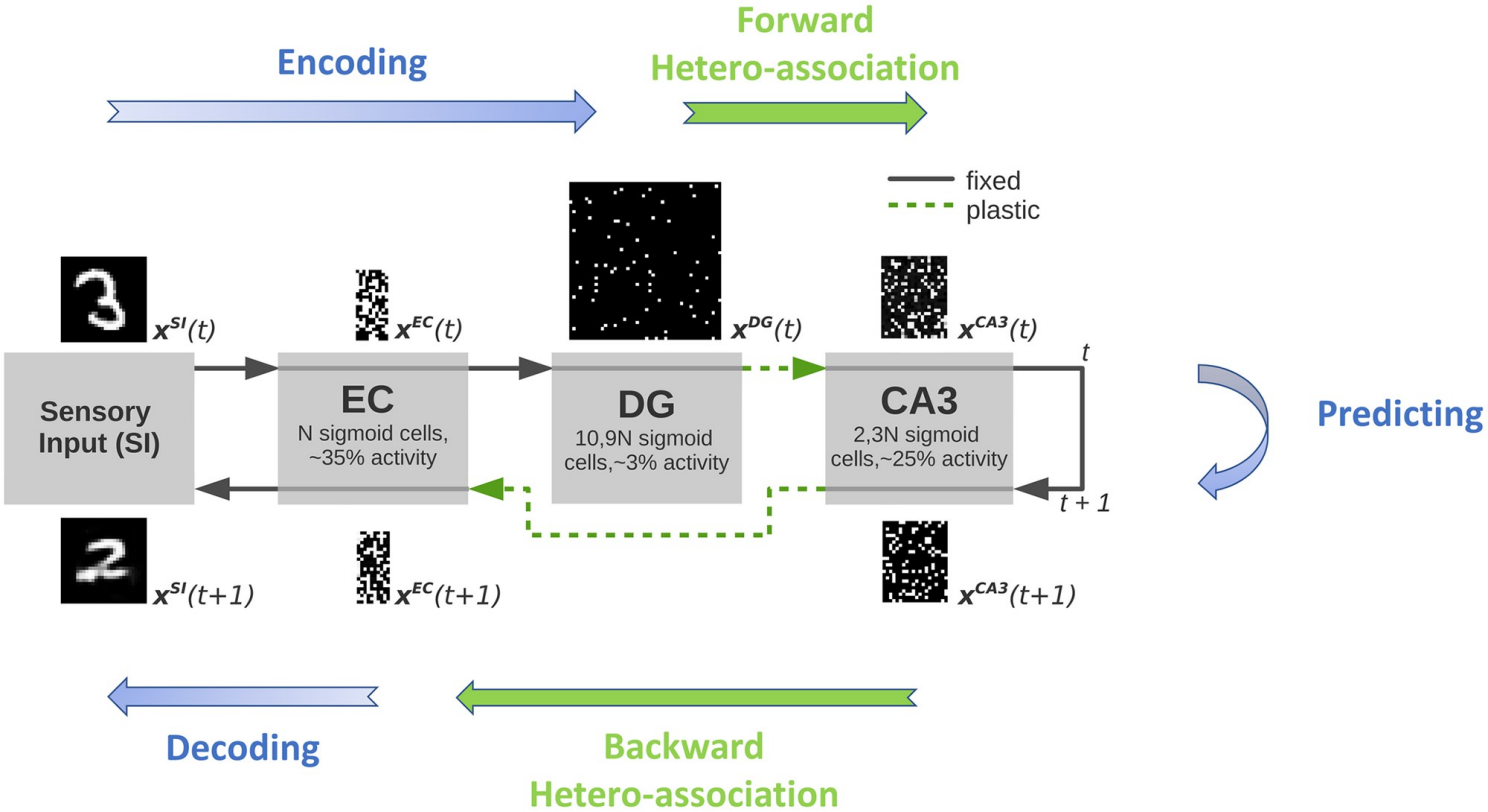
Illustration of the hippocampus model. The sensory input pattern **x**^*SI*^(*t*) is transformed to a binary representation through the mapping between SI and EC. DG then transforms the pattern **x**^*EC*^ to a sparse representation **x**^*DG*^ via the non-plastic pathway EC → DG which is subsequently propagated to CA3 via the plastic pathway DG → CA3. Here, *N* denotes the number of neurons in EC, and *t* denotes the time within the sequence. Dashed green arrows denote plastic pathways, while solid gray arrows denote fixed pathways, i.e. not plastic on the fast timescale of learning we consider in this work. The forward path represents the encoding path, while the backward path represents the decoding path. Prediction of the next pattern happens in CA3. For illustration purposes, example patterns in the corresponding subregions are shown.

Our model of the hippocampus includes the subregions EC, DG, and CA3 (Fig 1). It is used to store sequences of input patterns and retrieve them in the correct order given only a single cue pattern.

The model consists of an encoder part, a decoder part, and intrinsic dynamics in between. The encoder part of the network transforms the input sensory data **x**^*SI*^(*t*) to a binary representation **x**^*EC*^(*t*) and later to a sparse binary representation **x**^*DG*^(*t*). The decoder part of the network on the other hand transforms the binary pattern in EC back to the input SI for visualization purposes. Plasticity occurs in the DG → CA3 and CA3 → EC connections. The former weights hetero-associate a sequence of sparse binary patterns **x**^*DG*^(1), **x**^*DG*^(2), **x**^*DG*^(3), ⋯ with an intrinsic sequence of binary patterns **x**^*CA*3^(1), **x**^*CA*3^(2), **x**^*CA*3^(3), ⋯, one pattern pair at a time. The CA3 → EC weights perform a hetero-association in the reverse direction. The patterns in EC are either generated randomly for analysis purposes or, more realistically, sensory input is transformed via the SI → EC pathways to binary patterns in EC (Fig 1).

According to the standard framework, DG is believed to perform pattern separation that allows the association of very similar patterns in EC with different patterns in CA3. This is achieved here by a generic DG, pre-trained on random patterns independent of the input statistics. The non-plastic recurrent connections in CA3 are pre-trained to provide an intrinsic sequence of random patterns, which is described in more detail along with data generation, pre-training, and pattern storage/retrieval in the following sections.

The implementation of our models is based on the Python library PyDeep [28], which is publicly available at https://github.com/MelJan/PyDeep. The source code for model training, usage, and evaluation is made available under the same address (https://github.com/MelJan/OneShotHippocampus). All requested information and data for replication is available under this GitHub repository link.

### 2.2 Artificial neuron model

In this work, we consider rate-based models in which each neuron is represented by a centered artificial neuron [29, 30].

$$\begin{array}{c} h_{j}=\phi (a_{j})=\phi (\sum_{i}^{N}(x_{i}-\mu_{i})w_{ij}+b_{j}), \end{array}$$ (1)

with output activity *h*_*j*_, activation function *ϕ*(⋅), membrane potential *a*_*j*_, pre-synaptic input *x*_*i*_, offset value *μ*_*i*_ (usually the target activity of the input layer approximating the mean activity, see Fig 1), weight *w*_*ij*_, and bias *b*_*j*_ (see S1 Table in S1 Appendix for a full list of model and parameter descriptions). We use the sigmoid and step activation functions, but others could be used instead, which in our experience often leads to similar results. By centering, i.e. subtracting the mean from the input layer, the pre-synaptic activation becomes mean-free. Centering has been shown to be necessary for one-shot storage of patterns in artificial neural networks independently of the used learning rule [27]. Even in batch learning, although biologically not plausible, centering often leads to better results, especially in the case of unsupervised learning [30].

### 2.3 Learning rule

We use Hebbian descent as a learning rule for all subregions, which is a stable, and biologically plausible learning rule for hetero-association as well as auto-association [27]. Hebbian descent performs significantly better than Hebb’s rule and the covariance rule in general and significantly better than gradient descent in online learning. Furthermore, Hebbian descent provides an automatic mechanism of forgetting, see [27] for a detailed comparison of these methods. For hetero-associating an input activity *x*_*i*_ with a target output activity *t*_*j*_, updates for weight and bias are given by

$$\begin{array}{c} \delta w_{ij}=-\eta \left(x_{i}-\mu_{i}\right)\left(h_{j}-t_{j}\right), \end{array}$$ (2)

$$\begin{array}{c} \delta b_{j}=-\eta \left(h_{j}-t_{j}\right), \end{array}$$ (3)

with learning rate *η*. Hebbian-descent is therefore based on the correlation between the network’s centered input (*x*_*i*_ − *μ*_*i*_) and the difference between the network’s produced output *h*_*j*_ and target output *t*_*j*_. In the case of auto-association, the reconstructed input is given by the equation:

$$\begin{array}{c} z_{i}=\phi (\sum_{j}^{M}(h_{j}-\lambda_{j})w_{ij}+c_{i}), \end{array}$$ (4)

with hidden bias *c*_*i*_, and the updates for weight and bias are given by

$$\begin{array}{c} \delta w_{ij}=-\eta \left(h_{j}-\lambda_{j}\right)\left(z_{i}-x_{i}\right), \end{array}$$ (5)

$$\begin{array}{c} \delta c_{i}=-\eta \left(z_{i}-x_{i}\right), \end{array}$$ (6)

$$\begin{array}{c} \delta b_{j}=-\eta \left(h_{j}-{\tilde{\lambda }}_{j}\right),\;\text{(optional)} \end{array}$$ (7)

with learning rate *η*, hidden offset λ_*j*_ (i.e. the mean of the output unit *j*), and a target average hidden activity ${\tilde{\lambda }}_{j}$. Eq 7 is optional because the difference between the output activity *h*_*j*_ and the average hidden activity ${\tilde{\lambda }}_{j}$ are both close to zero since the activities are centered, and therefore the change is negligible. Notice, that analogously to contrastive Hebbian learning [31] this update rule is only local if we introduce time such that **x** and **z** are the post synaptic activities of the same neurons at two different time steps. Notice that the learning rate here could be tweaked to control plasticity; reducing it decreases learning/storage and if set to zero that completely suppresses the learning in the network.

### 2.4 Model assumptions and simplifications

For simplicity, we stored only one very long sequence of length *N* rather than multiple shorter sequences in the model. This is only to simplify the analysis, one could equivalently store a larger number of shorter intrinsic sequences in CA3 (see also [19]). The model just would have to stop encoding and retrieval after a smaller number of sequence elements.

We did not distinguish between the deep and superficial layers in EC, consistent with experimental evidence that the layers in EC act in unity [32]. Notice that this does not mean (unless explicitly mentioned in the simulations) that the output activities in EC are projected back to the current input in EC, as the input and output pathways through EC were considered at different time points in any case. Furthermore, in our simulations, we saw little to no effect on the quality of the retrieval of patterns in EC when the hippocampal subregion CA1 was also incorporated into the model in the decoding pathway. We therefore did not include CA1 for simplicity.

In the model, the number of neurons in the subregions is given relative to *N*, the number of neurons in EC, specifically, CA3 consisted of 2.3*N* neurons and DG of 10.9*N* neurons. The relative sizes of the subregions correspond to anatomical data (see [33], Fig 1). *N* = 100, 000 would lead to a model of the size of the rat hippocampus. Since this is too expensive computationally, we consider models of significantly smaller size (*N* = 200 and *N* = 1000).

Since the network model was 100 or 500 times smaller than the rat hippocampus, we correspondingly scaled up the average activity, i.e. the proportion of cells that are active on average at any given time, compared to recordings from the rat hippocampus (see [33], Table 1). That is, 25% instead of 3.2% for CA3 and 3% instead of 0.78% for DG. If we used an average activity 3.2% for *N* = 100, there would be only eight active CA3 neurons on average. This number of active neurons is too low for robust pattern association. As will be shown in the simulations, the model works with sparser activity levels as it becomes larger without reducing the capacity, so that a model of realistic size will most likely work with realistic activity levels. Furthermore, we will show that the required activity level also depends on the type of input data.

We considered an all-to-all connectivity between the subregions, even though the number of connections is much lower according to anatomical recordings from the rat hippocampus (see [33], Fig 1). In our experience, the choice of a reduced fixed network connectivity, e.g. random, locally, etc. has a significant impact on the network performance especially for smaller models, and might thus lead to misleading conclusions when analyzing the entire system. We thus preferred to use an all-to-all connectivity between the subregions and let the results determine the optimal connectivity to use. In addition, as argued above, we also assume that larger networks can do with sparser connectivity, while smaller networks need to have more complete connectivity in order to convey sufficient information.

## 3 Training the hippocampus model

### 3.1 Training of the individual subregions

Training is separated into two phases; the pre-training of individual subregions, which is done offline, and the one-shot storage phase, which is online. In the following subsections, the training of the specific subregions and the one-shot storage phase are described. A detailed network description is given in S1 Appendix.

#### 3.1.1 Pre-training CA3 as an intrinsic sequence generator

A stable and robust sequence generation in CA3 is crucial for the performance of the entire system. If the intrinsic dynamics would not return the correct following pattern **x**^*CA*3^(*t* + 1) given **x**^*CA*3^(*t*), the model would not be able to retrieve the correct sequence of patterns in CA3 and consequently also not the correct outputs in EC. Patterns in the intrinsic sequence in CA3 should thus not be too similar, as they could easily interfere with each other. Hence, they should ideally be orthogonal to each other or at least have a large pairwise Euclidean distance [19]. We therefore pre-trained CA3 on a cyclic sequence of *N* independently sampled binary random patterns **x**^*CA*3^(1), **x**^*CA*3^(2), ⋯, **x**^*CA*3^(*N*), where each random pattern has an average activity of 25%, unless otherwise mentioned. We used the hetero-associative Hebbian descent update (Eqs 2 and 3), where in each update step the input corresponds to one of the patterns **x**^*CA*3^(*t*) and the target output corresponds to the following pattern **x**^*CA*3^(*t* + 1). We used a learning rate of 1.0 and a mini-batch size of 10, i.e. the update was calculated on the average of the Hebbian decent learning rule over 10 samples. Pre-training of CA3 was performed for 100 epochs, i.e. each pattern was presented to the network 100 times to improve learning. In each epoch we randomly flipped 10% of the input values to induce more robustness with respect to noise in the input (multiple epochs were only used during pre-training, otherwise learning was online and in one-shot). At an average activity level of 25%, the offset values were *μ*_*i*_ = 0.25. Theoretically, we can store any set of 2.3*N* patterns perfectly in our model, e.g. when each pattern in CA3 is a one-hot encoded vector. The theoretical capacity of our model is therefore at least *C* = 2.3*N*. Note that depending on the statistics of the input and CA3 patterns the true capacity might be higher than this, but it is the guaranteed theoretical lower bound for any set of patterns.

#### 3.1.2 Pre-training DG as a pattern separator

Our DG model was generic in that it was independent of the actual input statistics and was the same for all datasets. This was achieved by training the connections EC → DG using the Hebbian descent update in Eqs 5–7 on random binary data. It can formally be seen as training an auto-encoder with tied weights and sigmoid non-linearity. Tied weights mean that the decoder weights are the transpose of the encoder weights, which is a way for parameter sharing. The hidden representation, which corresponds to DG, was regularized to have a hidden activity of approximately 3% by setting hidden offsets λ_*j*_ as well as the target activities ${\tilde{\lambda }}_{j}$ to 0.03, unless mentioned otherwise. The network was trained on 4000 random patterns with an EC activity level of 35%, so that *μ*_*i*_ is set to 0.35. We used a single epoch and a mini batch size of 10 resulting in 400 updates using a very large learning rate of 100. After training, the encoder part of the auto encoder, i.e. EC → DG) was used to transform any pattern with an activity of 35% to a 10.9 × larger DG pattern with an activity level of approximately 3% (see [27] for a detailed illustration of auto-encoders trained with Hebbian descent). Note that a network trained in this way is much more robust with respect to noise compared to a network with randomly initialized weights. A similar effect has been observed for recurrent networks [19].

#### 3.1.3 Pre-training the transformation of the sensory input

When sensory input data was provided, we trained the pathways SI → EC and EC → SI on the entire training data using the auto-associative Hebbian descent update given by Eqs 5–7. This can be formally seen again as training an auto-encoder with tied weights and a step function as output non-linearity to transform the potentially real-valued input data into a binary representation. The visible offset *μ*_*i*_ is set to the mean of the dataset and by setting the hidden offset λ_*j*_ as well as the target activities ${\tilde{\lambda }}_{j}$ to 0.35, we ensure that the activity level in EC is approximately 35%. We used a mini-batch of size 100, a learning rate of 0.01, and a momentum (fraction of the historical update added to the current update) of 0.9 for 10 epochs on the entire training data. After training, the encoder part of the network (Eq 1) transforms an input data-point **x**^*SI*^(*t*) to a binary representation **x**^*EC*^(*t*) with an activity level of approximately 35% and later through DG to a larger sparse binary representation. The decoder part of the network (Eq 4), which transforms the binary pattern in EC back to the input SI, can then be used to visualize the retrieved patterns in the input domain.

#### 3.1.4 One-shot storage in plastic pathways

For training the hetero-associative pathways DG → CA3, and CA3 → EC we use the hetero-associative Hebbian descent update (Eqs (2) and (3)). Here, we used the online version to perform one-shot learning, meaning that each pattern pair was available to the network only once and there was only one update step per pattern pair. For the DG → CA3 pathway for example, **x**^*DG*^(*t*) was the input and **x**^*CA*3^(*t*) was the target output pattern. The input offsets *μ*_*i*_ are initialized to the average activity of the corresponding subregions, i.e. 0.03 for DG → CA3. Although a constant learning rate of 0.1 works fairly well, a learning rate that takes the network size into account performs better in our experience. We found empirically that the simple learning rate

$$\begin{array}{c} \eta =\frac{20}{\text{N}} \end{array}$$ (8)

works well in practice for networks of size *N* = 20 to 2000. Since Hebbian descent controls the gradual forgetting of patterns automatically, we do not need a weight decay for stability as required, for example, when using Hebb’s rule.

## 4 Simulations setup

### 4.1 Input sequences

Patterns in EC were either generated randomly or represented real-world data that has been transformed to a binary representation using the SI → EC pathway. We have generated two artificial datasets, the RAND and the RAND-CORR datasets. The RAND dataset consists of uncorrelated random binary patterns, where 35% of the pixels are randomly selected and set to one and the remaining 65% of the pixels are set to zero, which is in line with the average activity in EC. The second dataset RAND-CORR contains random binary patterns that are temporally correlated and are generated as follows: In the first step, an initial pattern is generated where 35% of the pixels are set to one, and the rest are set to zero. Every subsequent pattern is generated by starting with the preceding pattern, from which we selected 10% of all pixels, half of them with value one and half of them with value zero, and flipped their values. While the average correlation of the patterns in the dataset is close to zero, pairs of subsequent patterns have a high correlation of 0.8.

For real world data, the well-known MNIST dataset was used, which consists of 70,000 grayscale images of handwritten digits divided into a training and test set of 60,000 and 10,000 patterns, respectively [34]. The images have a size of 28 × 28 pixels, where all pixel values are normalized to lie in a range of [0, 1]. The dataset is not binary, but the values tend to be close to zero or one. Each pattern is assigned to one out of ten classes, representing the digits 0 to 9.

An input sequence for the models was generated by selecting *N* patterns of the training data of one of the datasets.

### 4.2 Evaluation

We used the Pearson correlation coefficient as a performance measure, which is common in computational neuroscience [19, 33]. The higher the correlation between the retrieved pattern $\dot{\text{x}}$ (not to be confused with time derivative) and the ground truth pattern **x**, the better the performance. In each subregion, we calculate:

$$\begin{array}{c} \text{Corr}\left(\dot{\text{x}},\text{x}\right)≔\frac{\left\langle \left(\dot{\text{x}}-\left\langle \dot{\text{x}}\right\rangle \right)\left(\text{x}-\left\langle \text{x}\right\rangle \right)\right\rangle }{\sqrt{\left\langle {\left(\dot{\text{x}}-\left\langle \dot{\text{x}}\right\rangle \right)}^{2}\right\rangle }\sqrt{\left\langle {\left(\text{x}-\left\langle \text{x}\right\rangle \right)}^{2}\right\rangle }}, \end{array}$$ (9)

where the angular brackets 〈〉 indicate averaging over all patterns. Using the absolute error or squared error as a performance measure leads to qualitatively similar results. For non-artificial datasets, we additionally show the reconstructed patterns to visualize the performance of the network.

### 4.3 Storage and retrieval

**Fig 2:**
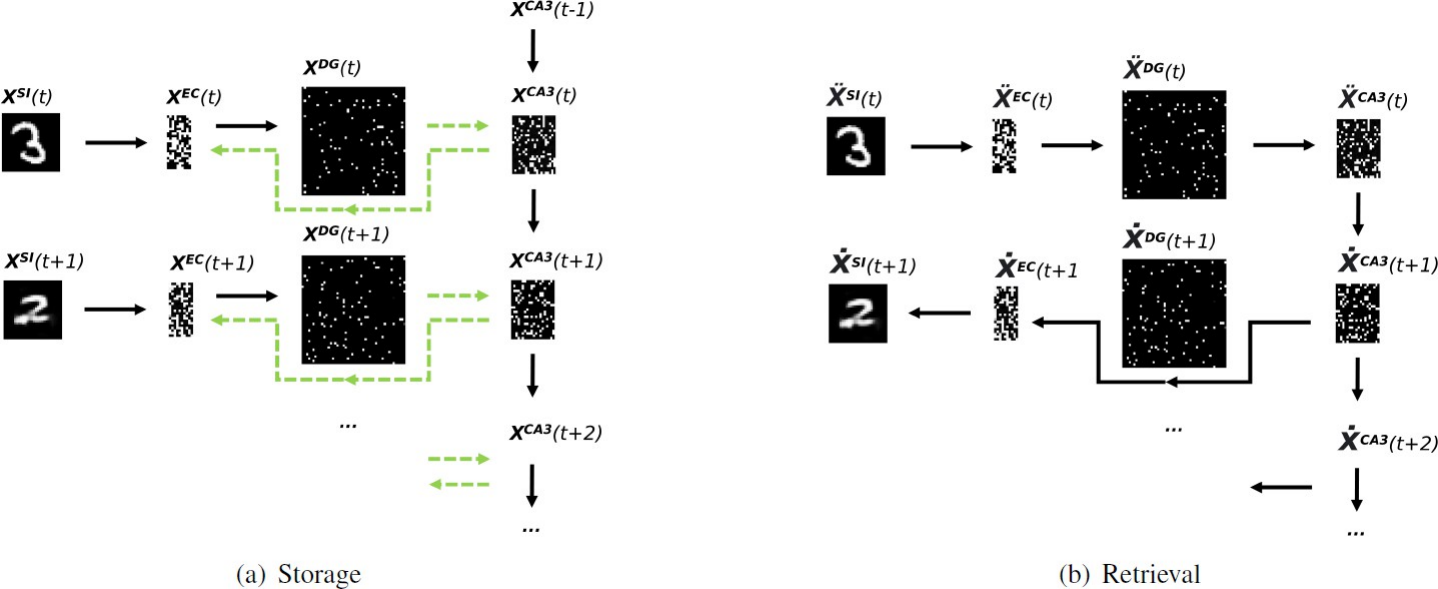
Illustration of (a) storage and (b) retrieval of a pattern sequence in our model. Solid black arrows denote propagation of activities, while dashed green arrows denote hetero-association in the corresponding pathway. Notice that the very first pattern **x**^*CA*3^(*t* − 1) is picked randomly from the intrinsic sequence and that ${\ddot{\text{x}}}^{SI}\left(t\right)$ denotes a potentially corrupted input cue pattern, and later ${\dot{\text{x}}}^{SI}\left(t+1\right)$ denotes a potentially corrupted retrieved pattern. During storage, SI via EC to DG serves as an encoder, and EC to SI serves as a decoder, while from DG to EC3 and from CA3 to EC is just hetero-association. However during retrieval, from SI to CA3 is encoding, while from CA3 via EC to SI is decoding. And if we use artificial stimuli that are not defined by images but by activities in EC, the encoding or decoding between SI and EC is discarded.

We consider storage and retrieval as two separate modes (Fig 2). During storage, the current input pattern **x**^*SI*^(*t*) is transformed via the SI → EC pathway to the pattern **x**^*EC*^(*t*) and then to **x**^*DG*^(*t*) (Fig 2(a)). At the same time, the intrinsic dynamics in CA3 generates an intrinsic pattern **x**^*CA*3^(*t*) from its predecessor **x**^*CA*3^(*t* − 1). Note that the CA3 dynamics is cyclic, so that each pattern has a unique predecessor, and that the very first pattern **x**^*CA*3^(*t* − 1) is picked randomly from the intrinsic sequence. Pattern **x**^*EC*^(*t*) and **x**^*CA*3^(*t*) are then hetero-associated in both directions through pathways DG → CA3 and CA3 → EC. In the next time step, **x**^*CA*3^(*t*) triggeres the next intrinsic pattern **x**^*CA*3^(*t* + 1), which is hetero-associated in both directions with pattern **x**^*EC*^(*t* + 1), and so on. We assume that the hippocampal theta rhythm suppresses the input from pathway DG → CA3, so that the CA3 states only depend on the intrinsic dynamics during encoding [35–37]. As mentioned, the intrinsic patterns are decorrelated and therefore they are unique; they look different from each other and look also different from the input patterns they are associated with.

On the other side, retrieval is initiated by providing a potentially corrupted cue pattern ${\ddot{\text{x}}}^{SI}\left(t\right)$, which is propagated via the SI → EC → DG → CA3 pathway (Fig 2(b)), while the intrinsic dynamics in CA3 is suppressed [36, 37], so that a pattern ${\dot{\text{x}}}^{CA3}\left(t\right)$ is triggered that should ideally be equal to the pattern **x**^*CA*3^(*t*) that has been associated with the input during encoding. After that, the DG → CA3 pathway is suppressed, so that the CA3 dynamics reactivates the intrinsic sequence starting from pattern ${\dot{\text{x}}}^{CA3}\left(t\right)$. Each intrinsic pattern is then back-projected into EC (and cortical areas), and for visualization purposes, even to the input level ${\dot{\text{x}}}^{SI}\left(t+1\right)$. This implements the main idea of CRISP [16], i.e. CA3 serves as an intrinsic sequence generator and plasticity occurs mainly in the feedforward connections of the hippocampal circuit. As shown in the simulations, this is a key requirement for recalling entire sequences from a single cue.

## 5 Results

In the following, simulation results will be illustrated. Here, we refer to the pathway (SI) → EC → DG → (CA3) as encoder, as it encodes the EC patterns into an intrinsic pattern. Similarly, we refer to pathway CA3 → EC → SI as the decoder and CA3 → CA3 as the recurrent connections which induce intrinsic dynamics. Whether SI is included in the encoder depends on whether we use images or artificial EC stimuli, and CA3 is included during recall, during the storage phase the DG → CA3 are not part of the encoder but hetero-associative. Please note that at the storage phase, the inputs to the model are the ground truth **x**^*SI*^(*t*), **x**^*EC*^(*t*), and **x**^*CA*3^(*t*) patterns, whereas at the recall phase the predicted ${\dot{\text{x}}}^{SI}\left(t\right)$, ${\dot{\text{x}}}^{EC}\left(t\right)$, and ${\dot{\text{x}}}^{CA3}\left(t\right)$ patterns are used instead.

### 5.1 Robust one-shot storage of correlated pattern sequences

**Fig 3:**
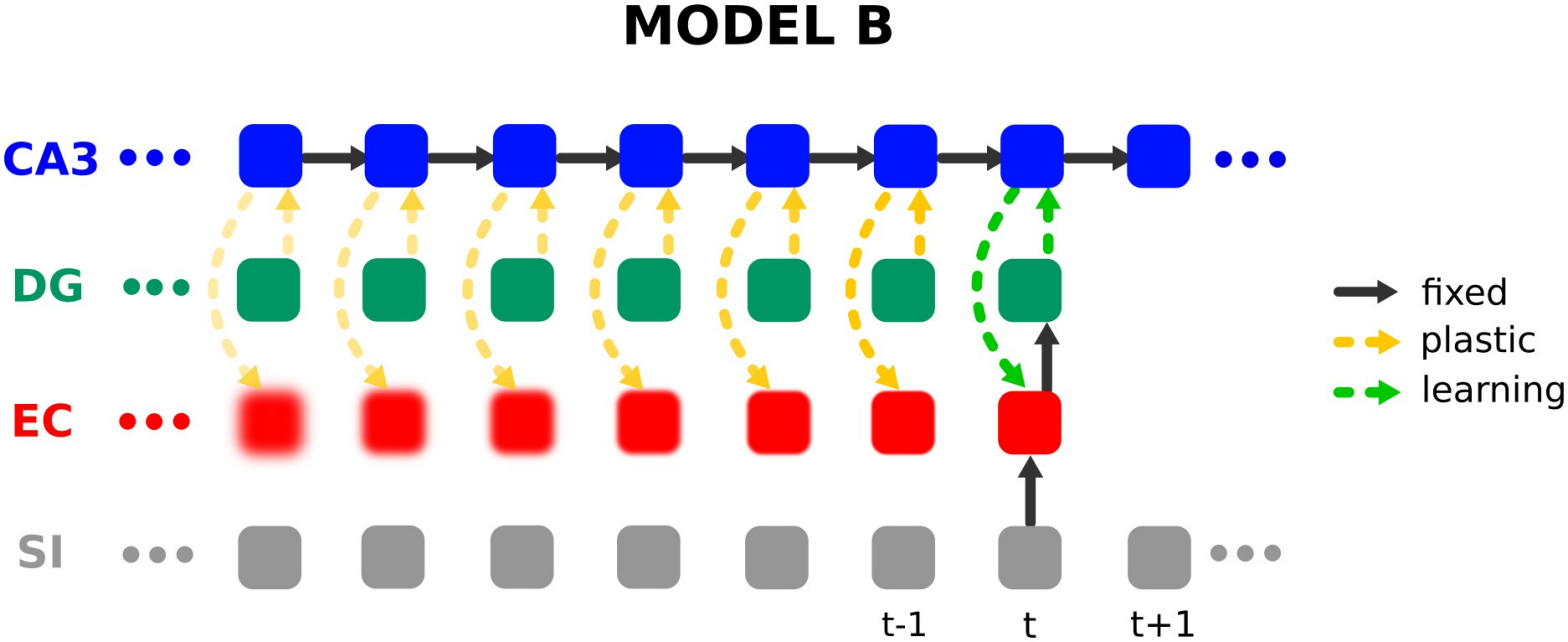
Illustration of hetero-association and forgetting over time in the hippocampus model. At time step *t*, the DG pattern, inferred via EC from the SI pattern, is hetero-associated with the CA3 pattern, which in turn is hetero-associated with the EC pattern (indicated by the green dashed arrows). The learned associations weaken over time (indicated by the increasing transparency of the orange arrows) leading to a degraded reconstruction (forgetting) in EC (indicated by the increasing blurring), which is stronger for remote patterns, like t-6, than for more recent patterns, like t-1. The figure illustrates those connections that are relevant to the current activity.

During storage in the Hippocampus model, the input patterns are propagated to DG and then hetero-associated with the intrinsic patterns in CA3. Notice that these associations weaken over time, which causes a degradation in the quality of recalled patterns for earlier stored patterns as will be shown in the next section. This is induced by the forgetting property of the Hebbian descent learning rule, and for clarity, we illustrate in Fig 3 how these associations are affected over time. Notice that since the intrinsic sequences are pretrained they are very reliable (indicated by the solid black arrows), thus it is to expect that the intrinsic sequence itself is very stable. However, the individual patterns within the sequence, which are associated to the patterns in the intrinsic sequence, degrade, the remote patterns more than the recent ones.

For the following results, we observe the performance of the HC-model for the recalled patterns after it has been trained on the *MNIST* dataset with *N* = 200. We also ran simulations using the *CIFAR* dataset which are not shown here but can be found in the arXiv preprint version of this paper [38].

#### 5.1.1 Visualization of input, reconstructed, and recalled patterns

**Fig 4:**
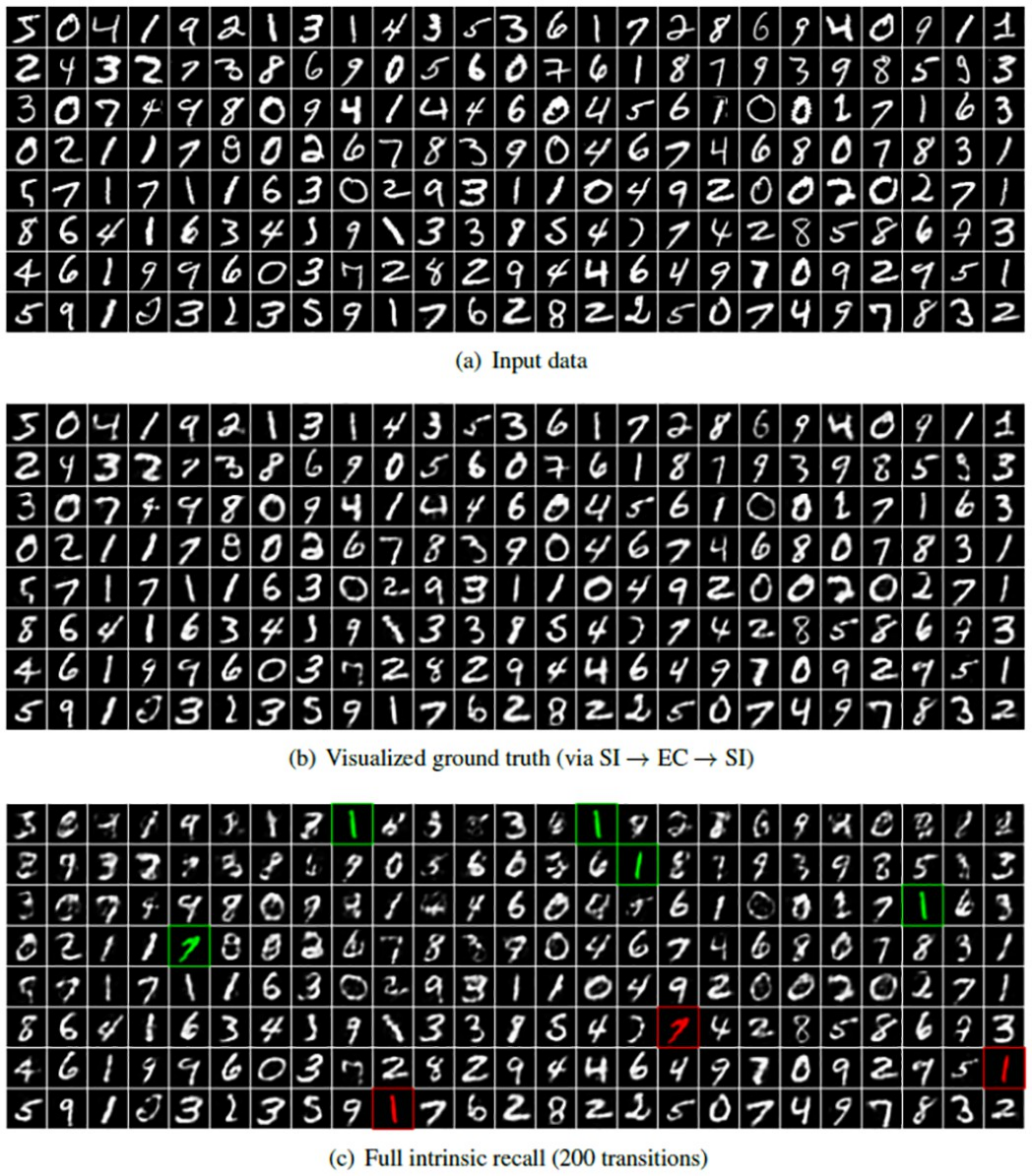
Visualization of *MNIST* sequences, which is read by rows left to right, top to bottom, so that the earliest pattern (index = 1) is located top left and the latest pattern (index 200) bottom right. (a) Sequence of input images as provided to SI. (b) Reconstruction of the same sequence encoded and directly decoded through SI → EC → SI (visualized EC ground truth). (c) Full intrinsic recall with 200 transitions for each pattern separately, which is visually very close to the reconstruction from the CA3 ground truth (CA3 → EC → SI, not shown). The patterns highlighted in green are examples of cues that have a doppelganger, highlighted in red, in the training sequence, and whose subsequence could be as a result recalled instead, especially for early (weakly remembered) patterns.

In order to get a more intuitive understanding of the performance of the system, reconstructed inputs and recalled patterns are visualized. When using real world data, the performance of the network is confounded by information loss due to the compression of the input images in SI into the EC representation. Therefore, we consider the EC representation as input ground truth, as we are not interested in the performance of the pathway SI → EC → SI, and visualize it by reconstructing the images from the EC representations. Fig 4(a) shows the entire input sequence of 200 *MNIST* patterns stored in the HC model with *N* = 200, and Fig 4(b) shows the sequence when it has been encoded into a 200 dimensional hidden representation (EC) and decoded back to input space (SI). As the 784 dimensional real valued *MNIST* patterns are compressed to 200 dimensional binary patterns there is some information loss leading to a visualized EC ground truth that is a smoothed version of the input data, which is the best to expect from any pattern retrieval in the network. Notice, that this information loss depends on the size of the model relative to the input dimensionality, so that in the case of *N* = 1000 the 1000 dimensional hidden representation leads to an almost perfect reconstruction (data not shown). Fig 4(c) shows the sequence of reconstructed EC patterns after full intrinsic recall, i.e., for each pattern the CA3 dynamics is cued by the digit of Fig 4(b), then CA3 runs through the dynamics 200 times to arrive back at the pattern that is then visualized as Fig 4(c). Each pattern has its own cycle through all 200 patterns. Please note that there is an ambiguity for very similar input patterns, so that the CA3 dynamics does not reliably start from the correct digit, but possibly from a doppelganger. While a doppelganger input pattern can stimulate two patterns in the intrinsic sequence, these two patterns do not look the same in the intrinsic sequence, but they rather get activated by the same or a similar input pattern. Once the intrinsic sequence is triggered, all patterns are unique and there is no cause of ambiguity anymore. The patterns retrieved should be equivalent or at least similar to the visualized EC ground truth patterns shown in Fig 4(b). A comparison with Fig 4(a) makes little sense here because that would include the information loss by the pathway SI → EC → SI, which is not relevant for the hippocampal model. The results are also visually indistinguishable from the reconstruction from the CA3 ground truth via CA3 → EC → SI (results not shown) except for the patterns we highlighted in green. Those input patterns have a doppelganger and map equally well to two or more similar intrinsic patterns in the training sequence, so that the system cannot distinguish them from one another and most likely recalls the pattern that has been stored later or most recently in the sequence (highlighted in red). Notice that each displayed pattern is the result of a full intrinsic recall with 200 transitions by itself independently of other displayed patterns, so that even if what was retrieved initially was the wrong pattern, after going the full cycle the system ends up at a pattern that looks like the correct one, and that is what is displayed here, however, every pattern in between was actually wrong. In the next section, a number of examples will be shown for a detailed illustration of what happens in between.

#### 5.1.2 Recall performance and sequence relaxation dynamics

**Fig 5:**
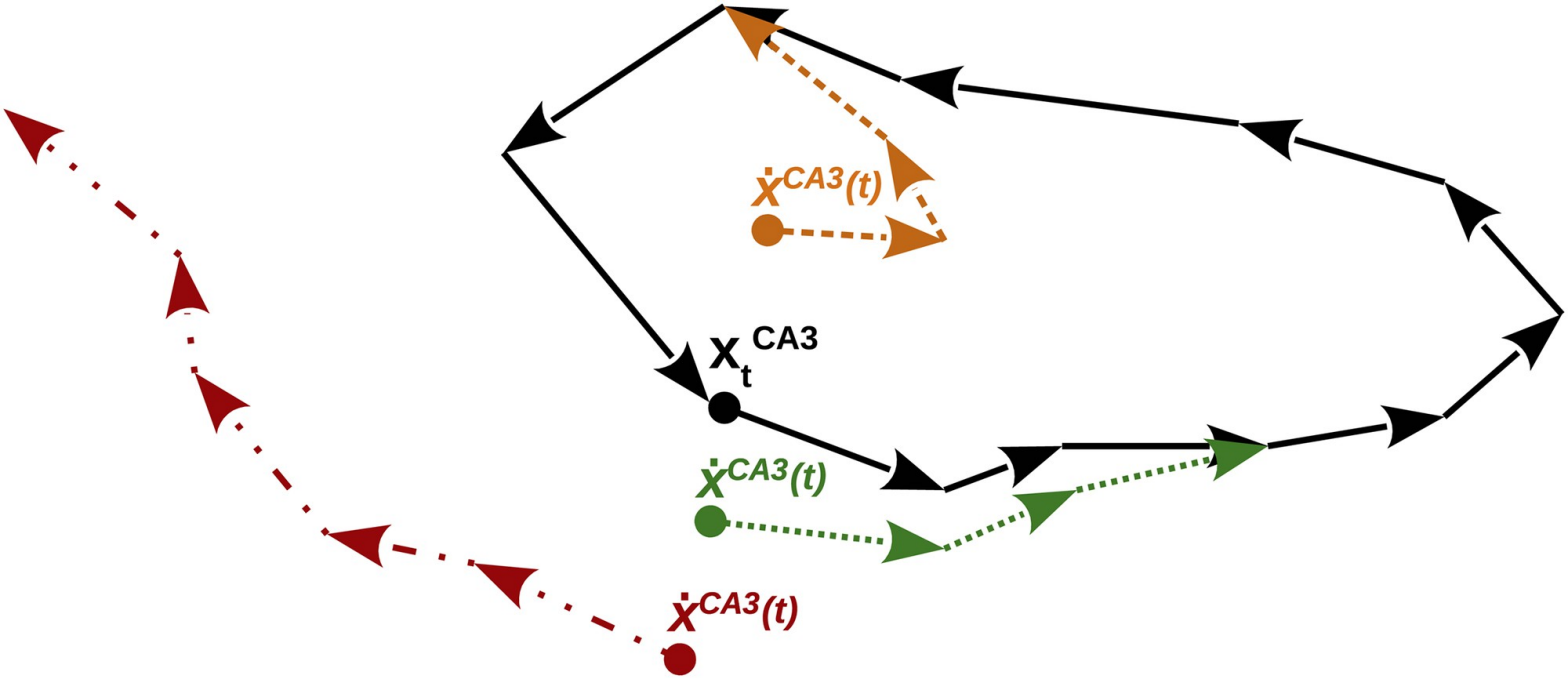
Illustration of the sequence relaxation scenarios in CA3. The solid black arrows represent the transitions between the patterns of the intrinsic sequence. If a corrupted cue is given to CA3 the system either (i) converges within a short transient to the right position in the intrinsic sequence if the cue is similar enough to the ground truth pattern (green dotted arrows), or (ii) converges to a wrong position in the intrinsic sequence if the cue is more similar to another pattern in the intrinsic sequence (orange dashed arrows), or (iii) converges to a spurious intrinsic sequence if the cue is similar to a pattern within a spurious sequence (red dash-dotted arrows).

After the storage phase, to investigate the model’s robustness to cue quality, a corrupted or noisy version of the input pattern that is similar to one of the patterns in the stored sequence can be fed to the HC-model. Three outcomes are here possible, firstly it can converge to the intrinsic sequence at the correct position, secondly it can converge to the intrinsic sequence but to a wrong position, and thirdly, which is the worst case, it can converge to a spurious state, where none of the retrieved patterns is similar to the corresponding ground truth pattern. Notice that in the two former cases, the CA3 dynamics needs a few iterations to converge on the intrinsic sequence exactly. The three possibilities of sequence relaxation in CA3, which depend on the cue quality, are illustrated in Fig 5.

#### 5.1.3 Visualization of individual recall subsequences

**Fig 6:**
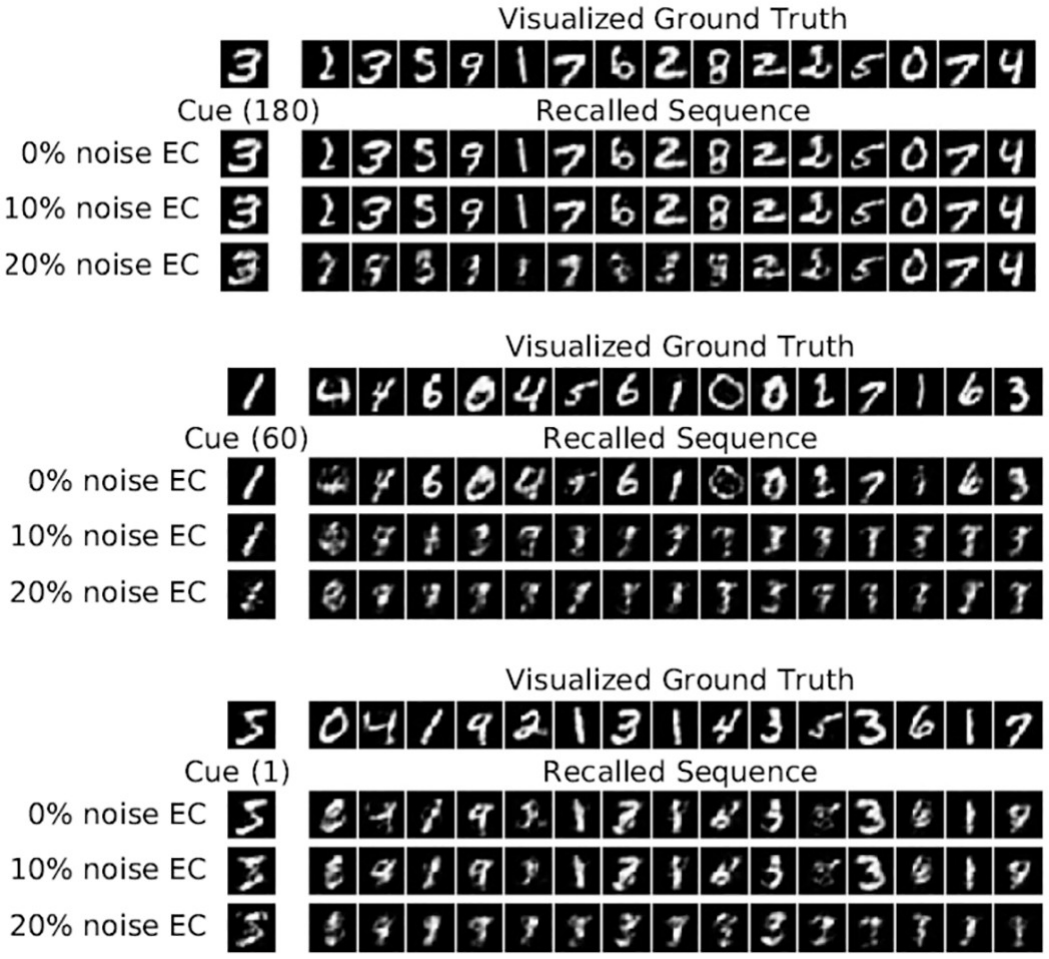
llustration of the recalled subsequences for different cues and noise levels for the HC model on the *MNIST* for *N* = 200. Each cue is propagated via pathway SI → EC → DG → CA3. The intrinsic transition is iterated for 15 transitions, in which in each transition the current pattern in CA3 is decoded via pathway CA3 → EC → SI to visualize the reconstructed digit pattern in SI. The first row shows the visualized EC ground truth cue and subsequence that have been decoded via pathway EC → SI to SI. The second row shows the reconstructions in SI when the exact pattern is presented in EC, and the remaining two rows show the results when either 10% or 20% binary noise is added to the corresponding EC pattern.

To further investigate the recall process, we show the first 15 patterns of the recalled subsequence for different cue patterns in Fig 6. The cues are taken from position 1 (early), 60 (middle), and 180 (late). The first row in each sub-figure shows the visualized EC ground truth subsequence starting with the cue pattern (*i.e*. the SI reconstruction from the ground truth EC patterns), which is thus a subsequence of the full sequence shown in Fig 4(b). The second row shows the cue and recalled subsequence when the exact cue with 0% noise has been presented and the remaining two rows show corrupted cues and their recalled subsequences, where either 10% or 20% binary noise has been added to the corresponding EC pattern (20% binary noise means 10 of the on units are flipped off, and 10 of the off units are flipped on). All visualized patterns have been reconstructed via pathway EC → SI. For all 0% noise cues, the system recalls the correct subsequence after a short transition phase. The system also succeeds when 10% noise is added, except for cue 60, which converges to a spurious sequence. For 20% noise, only cue 180 recalls the correct sequence, suggesting that the recall process is more noise-sensitive for earlier patterns. Furthermore, the recall quality decreases with decreasing pattern index, as expected from the degraded CA3 → EC → SI performance as shown in Fig 11(d) and 11(f). For 50% noise, the system generates a spurious sequence in almost all cases and thus does not recall any meaningful pattern in SI (data not shown).

#### 5.1.4 On the difficulty of the *MNIST* dataset

**Fig 7:**
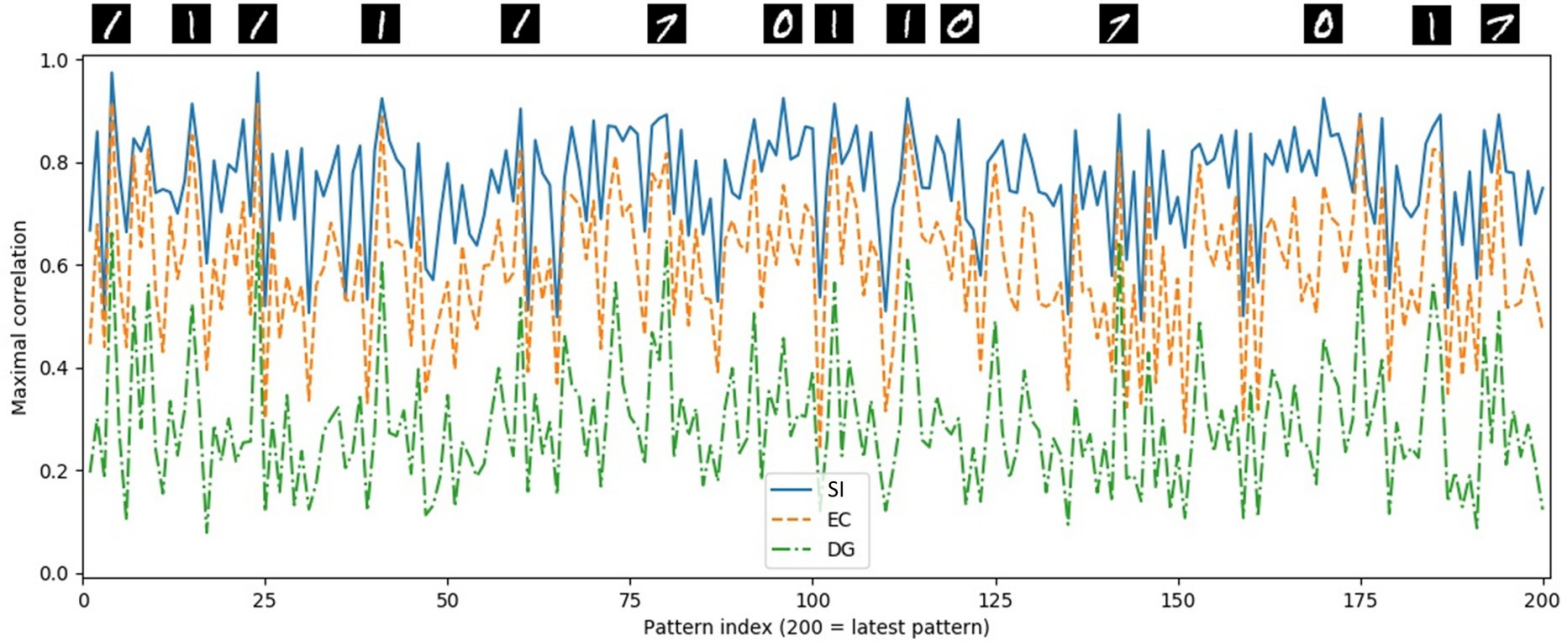
Maximum of the correlation between each pattern and all the other patterns in subregions SI, EC, and DG for the HC-model on the *MNIST* dataset for *N* = 200. For some of the peak values, the corresponding patterns are shown above the corresponding position (pattern index).

We have observed that for late patterns, the system achieves good recall, even in the presence of noise, whereas the robustness to noise reduces with decreasing pattern index. But there is a property of the *MNIST* dataset that also makes it sometimes harder for the model to relax to the correct position in the correct sequence in CA3. Compared to artificial datasets like the *RAND-CORR*, where we control the maximum correlation between two patterns in the input sequence to be approximately 0.8, the *MNIST* dataset has a much higher correlation for some of the patterns in the input sequence. This is illustrated in Fig 7, which shows the maximal correlation between each pattern and all the other patterns within the sequence for subregions SI, EC, and DG.

While the maximal correlation reduces only slightly when the patterns are propagated from SI to EC, it reduces significantly when propagated further to DG. However, the relations within the distributions stay relatively the same, meaning that a large correlation for a pattern in SI will still have a large correlation relative to the other patterns in EC and DG. We additionally show the patterns corresponding to the largest peaks, illustrating which patterns are most likely confused with another pattern in the sequence, namely two versions of the digit 1, one version of the digit 7 and one version of the digit 0. A recall of an input cue that has a doppelganger might initiate a recall of a subsequence that is close to the visualized EC ground truth subsequence of a similar but more recent pattern in the intrinsic sequence.

**Fig 8:**
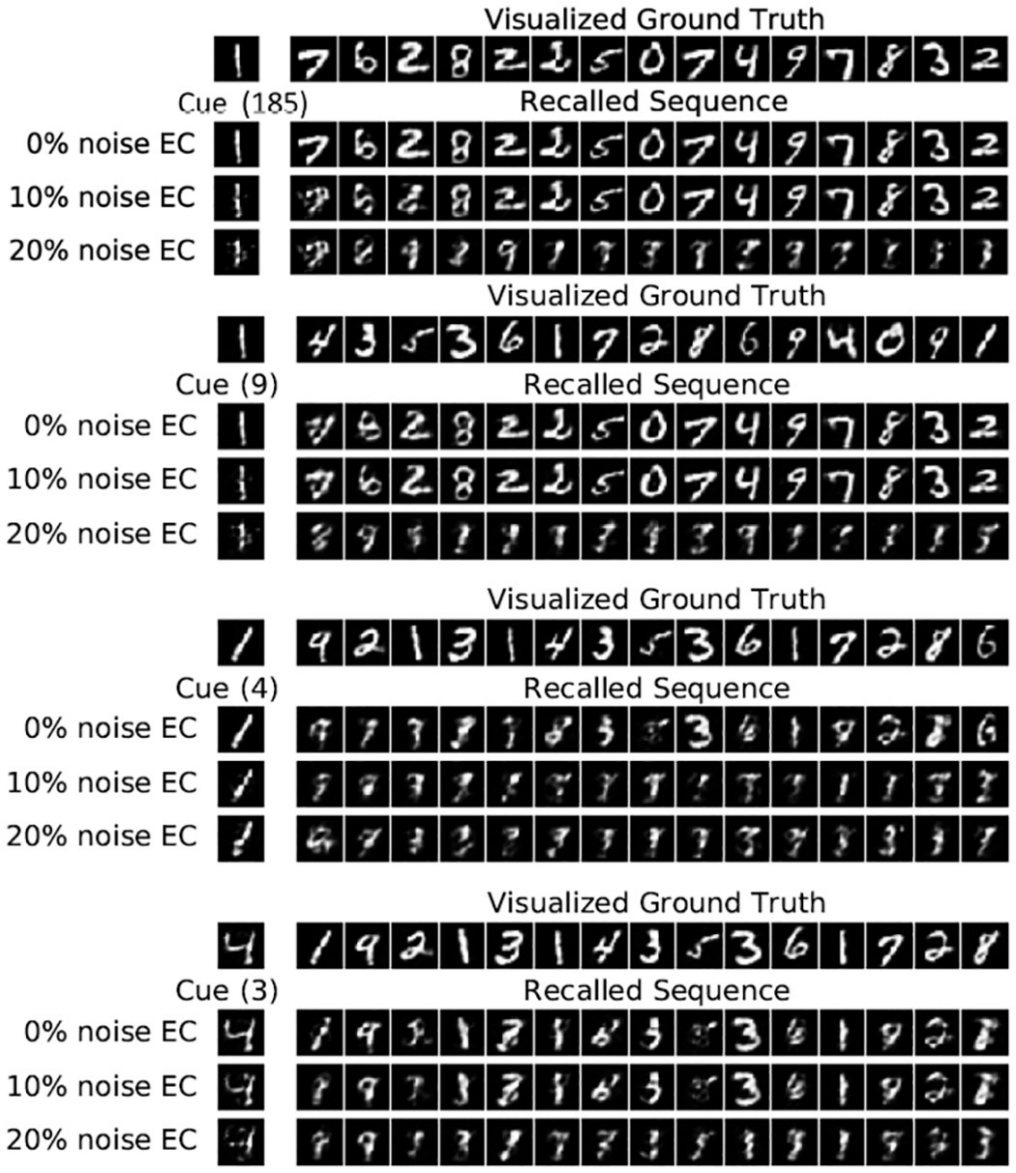
Illustration of recalled subsequences for different pairs of cues that are highly correlated on the *MNIST* for *N* = 200. Cue (185) with 0% noise is an example of correct relaxation and for relaxation to a spurious sequence with 20% noise. Cue (9) is an example of a relaxation to a wrong position (See Fig 5). Cue (3) does not have a doppelganger and recalls the correct subsequence, however Cue (4) has a doppelganger which causes a more degraded transient phase and needs more time to properly converge to the exact patterns of the intrinsic sequence.

The cue with index 9 is one of the five patterns highlighted in Fig 4 that has a doppelganger pattern with index 185. As shown in Fig 8, for these two similar patterns, even without noise, the system gets confused by a doppelganger and the subsequence that is recalled is the one for the more recent pattern with index 185, highlighted in red in Fig 4. This implies that if a pattern has a doppelganger in the dataset, it is more sensitive to noise because it can easily relax to the doppelganger’s subsequence. For 20% noise, the dynamics of cues 9 and 185 does not relax to either of the two subsequences within 15 intrinsic transitions. Moreover, in contrast to cue 3, shown in Fig 8, which does not have a doppelganger, cue 4 has a more degraded transient phase of about five patterns, because it has a doppelganger and needs more time to properly converge to the exact patterns of the intrinsic sequence. After five patterns the quality of the recall is more or less identical for cues 3 and 4, comparing the sequences shifted by one.

#### 5.1.5 Cue retrieval with novel input patterns

**Fig 9:**
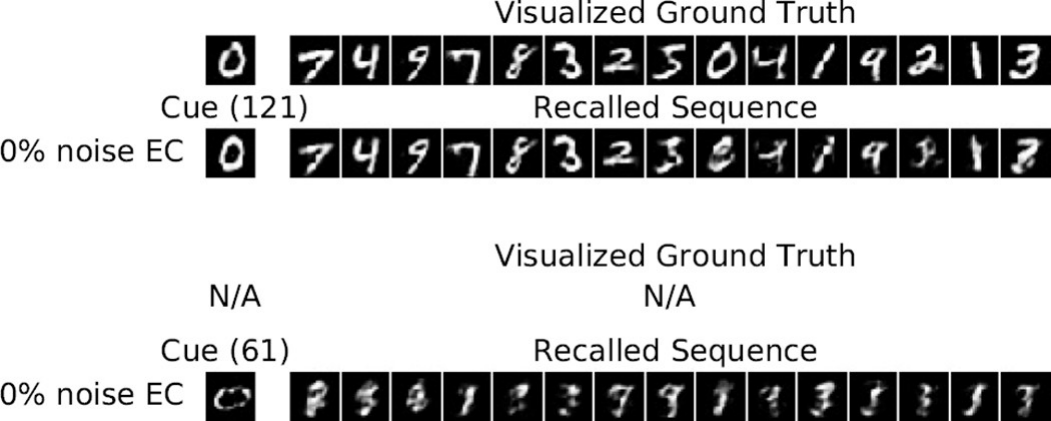
Illustration of the recalled subsequences, when cued with novel patterns (test data) for the HC-model on the *MNIST* for *N* = 200. For the first pattern, the stored sequence is recalled starting from a similar stored pattern, while for the second pattern, there exists no similar ground truth pattern and a spurious sequence is recalled.

We have seen that the model is robust to noise to some extent, but when the cue presented differs too much from the correct pattern the model does not recall any pattern of the visualized EC ground truth sequence. This is a desired property of the model as it should only recall the corresponding sequence when the presented cue pattern has a high similarity to the visualized EC ground truth cue pattern. To verify this we selected two patterns of the test dataset that the model has not seen before and presented them as cues to the network. One of those patterns is rather similar to at least one pattern in the training sequence while the other test pattern differs sufficiently from all patterns in the training sequence. The results are shown in Fig 9. The top row in the first two subfigures shows the manually determined ground truth sequence to which the recalled sequence, shown in the second row, is most similar. For the other cue there exists no similar ground truth as the recalled spurious sequence is significantly different from all shifted version of the ground truth sequence.

As illustrated, the HC-model can identify new unseen patterns as novel as long as they are different enough from all stored patterns. This could be used to implement novelty detection, which in agreement with other studies [39–41] we believe is performed by CA1. However, the role of CA1 is controversial, novelty detection could also be dependent on the DG and CA3 subregions [42]. Although the integration of subregion CA1 is postponed to future work, we want to discuss here how to train and integrate CA1 as a novelty/familiarity detector in our model. Instead of the CA3 → EC pathway, the CA3 → CA1→ EC pathway can be considered instead. The subregion CA1 could be trained in this case to identify whether the activity received from CA3 does or does not belong to the intrinsic sequence, and therefore perform novelty/familiarity detection on CA3/CA1 patterns, which had already been indicated by previous studies [39, 43]. Thus, when a test pattern such as the fourth pattern in Fig 9 triggers a spurious intrinsic sequence, CA1 would identify that the received activity from CA3 does not belong to a valid intrinsic pattern and send out a novelty signal. When the test pattern such as the first pattern in Fig 9 recalls the correct intrinsic sequence or at least very similar patterns, CA1 would identify this and send out a familiarity signal. Similar to CA3, CA1 can be pre-trained using Hebbian descent on the intrinsic sequence of CA3 and does thus not affect the online learning ability of the model.

### 5.2 Investigating the interplay between pattern separation and pattern completion

To study the role of DG in pattern separation and pattern completion in CA3, we use the artificial *RAND* and *RAND-CORR* datasets, in which the correlations between patterns are better controlled than in the real-world data.

#### 5.2.1 Pattern separation in DG is critical for encoding and sequence completion

**Fig 10:**
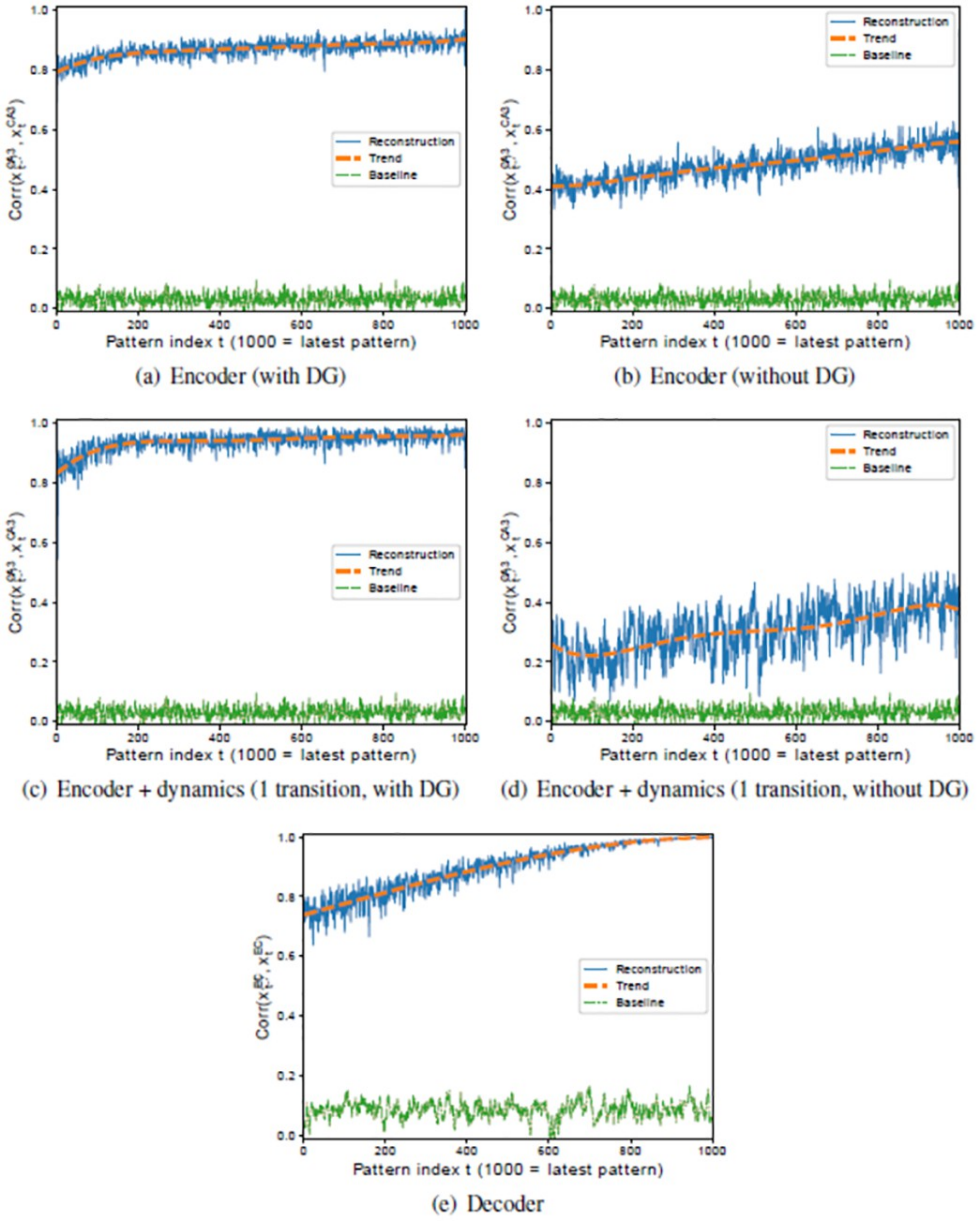
Encoding, intrinsic dynamics, and decoding performance on the *RAND-CORR* dataset with (left) and without (right) DG. The plots show the correlation between retrieved and ground truth patterns in CA3 or EC in solid blue. Additionally, a corresponding trend line is given in dashed orange as well as a baseline in dash-dotted green. The baseline denotes the correlation between retrieved patterns and the mean pattern of the entire sequence of ground truth patterns, i.e. $\text{Corr}({\dot{\text{x}}}^{CA3}\left(t\right),{\langle {\text{x}}^{CA3}\left(t\right)\rangle }_{t})$ and $\text{Corr}({\dot{\text{x}}}^{EC}\left(t\right),{\langle {\text{x}}^{EC}\left(t\right)\rangle }_{t})$, respectively. It thus reflects the trivial solution, if the network just generated the mean pattern as an output. (a) Encoder (${\text{x}}^{EC}\left(t\right)\to {\dot{\text{x}}}^{DG}\left(t\right)\to {\dot{\text{x}}}^{CA3}\left(t\right)$) (b) Encoder without DG (${\text{x}}^{EC}\left(t\right)\to {\dot{\text{x}}}^{CA3}\left(t\right)$). (c) Intrinsic performance in CA3, where each pattern is encoded and the intrinsic dynamic is iterated once (${\text{x}}^{EC}\left(t-1\right)\to {\dot{\text{x}}}^{DG}\left(t-1\right)\to {\dot{\text{x}}}^{CA3}\left(t-1\right)\to {\dot{\text{x}}}^{CA3}\left(t\right)$) (d) Intrinsic performance in CA3 excluding DG. (${\text{x}}^{EC}\left(t-1\right)\to {\dot{\text{x}}}^{CA3}\left(t-1\right)\to {\dot{\text{x}}}^{CA3}\left(t\right)$) (e) Decoder (${\text{x}}^{CA3}\left(t\right)\to {\dot{\text{x}}}^{EC}\left(t\right)$). In all plots, the input pattern is the ground truth, as well as the pattern that is compared to.

In the previous sections, we have shown that the model can recall sequences of correlated patterns from a single input pattern. To understand what drives this performance, we look at three key components that can perform pattern completion, see Fig 10. First, the input pattern is mapped to the high dimensional DG region before being hetero-associated with the pattern in CA3 (Fig 10(a)). Second, the CA3 intrinsic dynamics maps one element in the sequence to the next (Fig 10(c)). Third, the CA3 pattern is mapped onto the output in EC (Fig 10(e)). Note that we compare the ground truth patterns used during storage (**x**^*EC*^(*t*), **x**^*CA*3^(*t*)) with the recalled patterns (${\dot{\text{x}}}^{EC}\left(t\right)$, ${\dot{\text{x}}}^{CA3}\left(t\right)$). Each point represents the correlation between the reconstructed EC pattern and the retrieved cue after a full intrinsic recall, i.e., after the CA3 dynamics was iterated only once to arrive back at the pattern that was used as a retrieval cue. The correlation degrades gradually for patterns stored earlier (smaller pattern index), which indicates forgetting.

For clarity, we illustrated in Fig 3 how these associations weaken over time. The speed of forgetting is larger for the decoder than for the encoder, as it is easier to hetero-associate the very high dimensional patterns of DG with CA3 patterns than those with the low dimensional patterns of EC.

Pattern separation in DG is critical for encoding (Fig 10(a)), which is much worse if the DG is removed from the network and the EC pattern is propagated to CA3 directly instead (Fig 10(b)). This in turn makes it difficult for CA3 to trigger the right sequence (compare Fig 10(d) to 10(c)). As one might expect, the performance of the decoder is not affected by pattern separation in DG, since it is trained separately.

**Fig 11:**
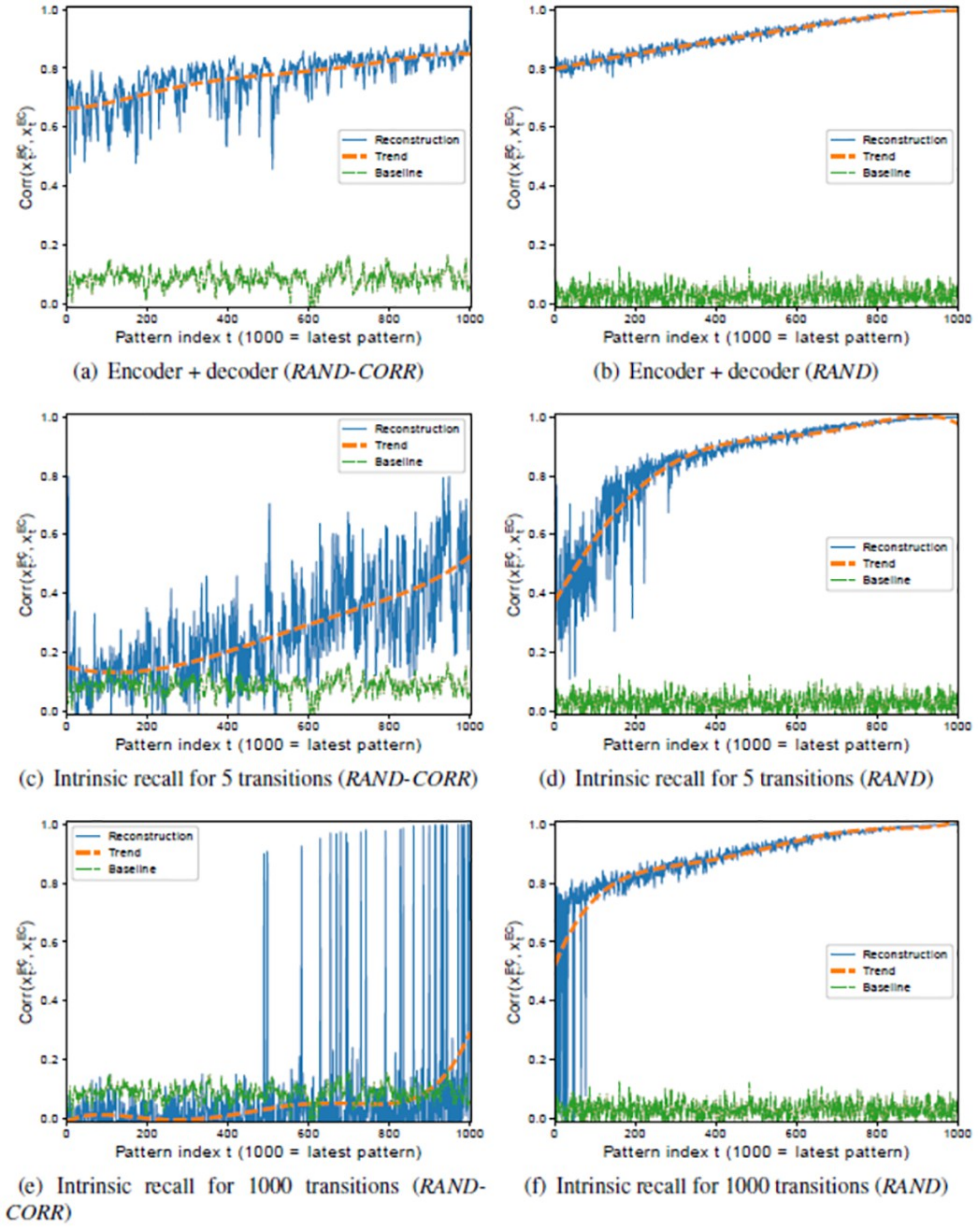
Recall performance of a simplified HC-model (excluding DG subregion) on the *RAND-CORR* (left) and *RAND* (right) datasets. (a,b) Each pattern is encoded and directly decoded without intrinsic dynamics, (${\text{x}}^{EC}\left(t\right)\to {\dot{\text{x}}}^{CA3}\left(t\right)\to {\dot{\text{x}}}^{EC}\left(t\right)$), for *RAND-CORR* and for *RAND* respectively. (c,d) Each pattern is encoded, the intrinsic transition is iterated five times, and the corresponding pattern is decoded, (${\text{x}}^{EC}\left(t-5\right)\to {\dot{\text{x}}}^{CA3}\left(t-5\right)\to {\dot{\text{x}}}^{CA3}\left(t-4\right)\to {\dot{\text{x}}}^{CA3}\left(t-3\right)\to \cdots \to {\dot{\text{x}}}^{CA3}\left(t\right)\to {\dot{\text{x}}}^{EC}\left(t\right)$), for *RAND-CORR* and for *RAND*, respectively. (e,f) Each pattern is encoded, the intrinsic transition is looped fully through (*T* transitions) arriving at pattern *t* again, and the corresponding pattern is decoded, (${\text{x}}^{EC}\left(t\right)\to {\dot{\text{x}}}^{CA3}\left(t\right)\to {\dot{\text{x}}}^{CA3}\left(t+1\right)\to {\dot{\text{x}}}^{CA3}\left(t+2\right)\to \cdots \to {\dot{\text{x}}}^{CA3}\left(T\right)\to {\dot{\text{x}}}^{CA3}\left(t+T-1\right)\to {\dot{\text{x}}}^{CA3}\left(t\;+\;T\;=\;t\right)\to {\dot{\text{x}}}^{EC}\left(t\right)$), for *RAND-CORR* and for *RAND*, respectively.

Even without a DG and even though the encoder performs poorly, the quality of the patterns in EC is surprisingly good when a pattern is encoded and directly decoded (Fig 11(a)), which shows that the error correction aspect of the decoder is able to compensate for the poor encoding. Even though the combination of encoding and decoding still works reasonably well, the performance in EC drops significantly with an increase in the number of intrinsic transitions (Fig 11(c)). The overall performance is near baseline for almost all patterns as the intrinsic dynamics cycles through the entire sequence (Fig 11(e)). In these cases, the CA3 dynamics either converges to a shifted version of the intrinsic sequence or to a spurious sequence. Notice that the intrinsic sequence in CA3 is cyclic, so that the successive pattern for **x**^*CA*3^(*T*) is **x**^*CA*3^(1).

A good performance of the encoder is hence crucial for the overall performance of the system, as all successive parts of the system depend on the encoder. Subregion DG, therefore, serves as a generic way of making sure that the input patterns are sufficiently decorrelated. By having the DG, the maximum correlation of 0.8 between two patterns in the dataset is reduced from 0.8 in EC to 0.45 in DG. This is similar to the results reflected in Fig 7 but in that case it is for the MNIST digits. Thus, it does not fully decorrelate the patterns, but it is enough to achieve a good performance of the encoder (EC → DG → CA3).

Another aspect, however, is the nature of the input stimuli. If there are no correlations in the input sequence, such as in the *RAND* dataset, even a model without DG does fairly well (Fig 11(f)). Here, increasing the number of intrinsic transitions actually improves the model performance and subsequently the quality of the recalled patterns. Already Bayati [19] have found that for offline learning (see [19] Fig 7 top left) uncorrelated patterns performance initially decreases and increases again as more intrinsic transitions are performed. While in offline learning all patterns are stored at the same time, and therefore the denoising property of CA3 is equally important for all patterns, in online learning we have shown that it is less important for recently stored patterns and becomes more important for older patterns. Moreover, due to the difficulty of online learning, the empirical capacity is lower, *C* ≈ *N*, and therefore $\frac{N}{2.3N}\approx 0.43$ of the theoretical capacity.

#### 5.2.2 Storing sequences through plasticity in CA3

**Fig 12:**
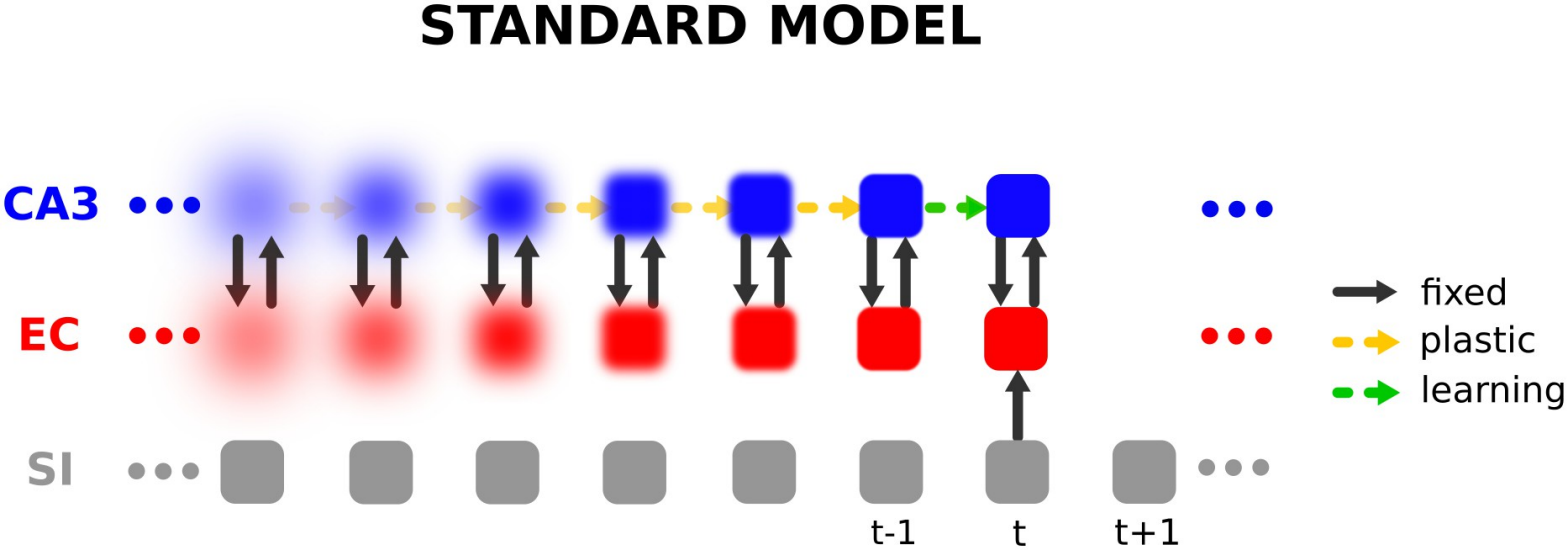
Illustration of hetero-association and forgetting over time in the standard framework. At time step *t* the current EC pattern (inferred from the corresponding SI pattern) is projected to CA3 via a fixed pathway EC→CA3 (here we simply used the identity mapping) and hetero-associated (indicated by the green dashed arrows) with the previous CA3 pattern. The learned associations weaken over time indicated by the increasing transparency of the arrows, leading to a degraded reconstruction (forgetting) in CA3 and thus also in EC indicated by the increasing blur.

In the standard framework [14] plasticity is assumed in the recurrent connections of CA3, so that online storage of sequences is performed by hetero-associating the previous with the current pattern in CA3 one pattern pair at a time [44]. This process is illustrated in Fig 12. Our model, in contrast, hetero-associates patterns to an already existing sequence which works better than storing a sequence in the Hippocampus by adapting the recurrent connections.

**Fig 13:**
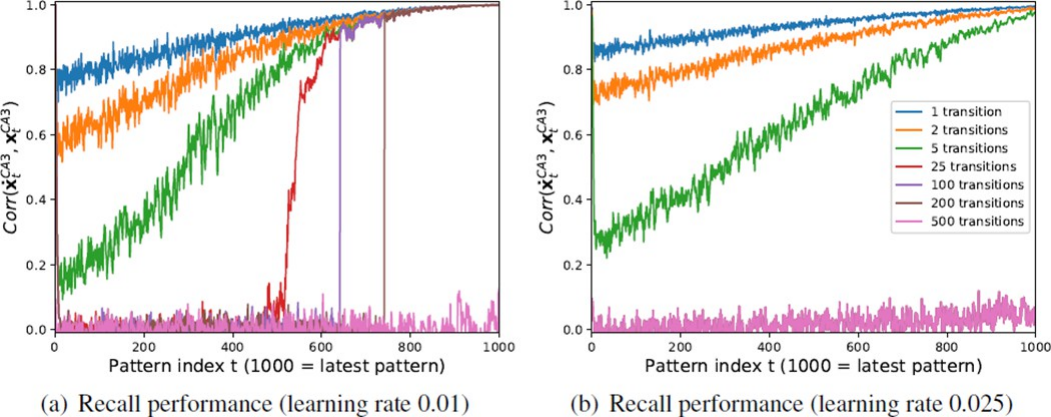
Recall performance in the standard framework, where uncorrelated CA3 patterns have been hetero-associated online one pattern pair at a time with a learning rate of (a) 0.01 and (b) 0.025. Shown is the network performance after a varying number of iterations through the recurrent CA3 dynamics (e.g. for 5 transitions ${\text{x}}^{CA3}\left(t-5\right)\to {\dot{\text{x}}}^{CA3}\left(t-4\right)\cdots {\dot{\text{x}}}^{CA3}\left(t\right)$). Notice, that the curves for 25, 100, and 200 transitions in (b) are almost perfectly overlaid by the curve for 500 transitions and thus not visible.

To illustrate this, simulations on online hetero-association of uncorrelated patterns were performed one pattern pair at a time, using the same CA3 sequence with the same dimensionality and activity as in the previous simulations. The recall performance for a different number of transitions was also evaluated, *i.e*. asking the question how well a pattern following a starting pattern after 1, 2, 5, 25, … time steps can be recalled. The results are shown in Fig 13(a), which shows that as the number of transitions increases, the performance decreases rapidly, so that recalling patterns after 25 transitions only works for the most recent third of patterns and recalling for 500 transitions and more does not work for any of the patterns. While the performance for this model decreases rapidly with the number of transitions, the performance for our model increases with the number of iterations as the model is able to recover through the sequence relaxation process in CA3. Notice, that the process is also very sensitive to the learning rate as illustrated in Fig 13(b). Using a learning rate larger than 0.025 or less than 0.01 leads to a much worse performance (results not shown). This provides evidence that the concept of intrinsic sequences as proposed by the CRISP framework might be superior to the standard framework, as online hetero-association of patterns in recurrent CA3 synapses leads to poor performance. It is important to note here, that our model also needs plasticity in CA3, however not during the storage phase but during the establishment of the intrinsic sequences phase that occurred earlier. For one-shot sequence learning, we believe it is not necessary, and therefore we don’t model it at this phase.

#### 5.2.3 Network activation level in CA3

**Fig 14:**
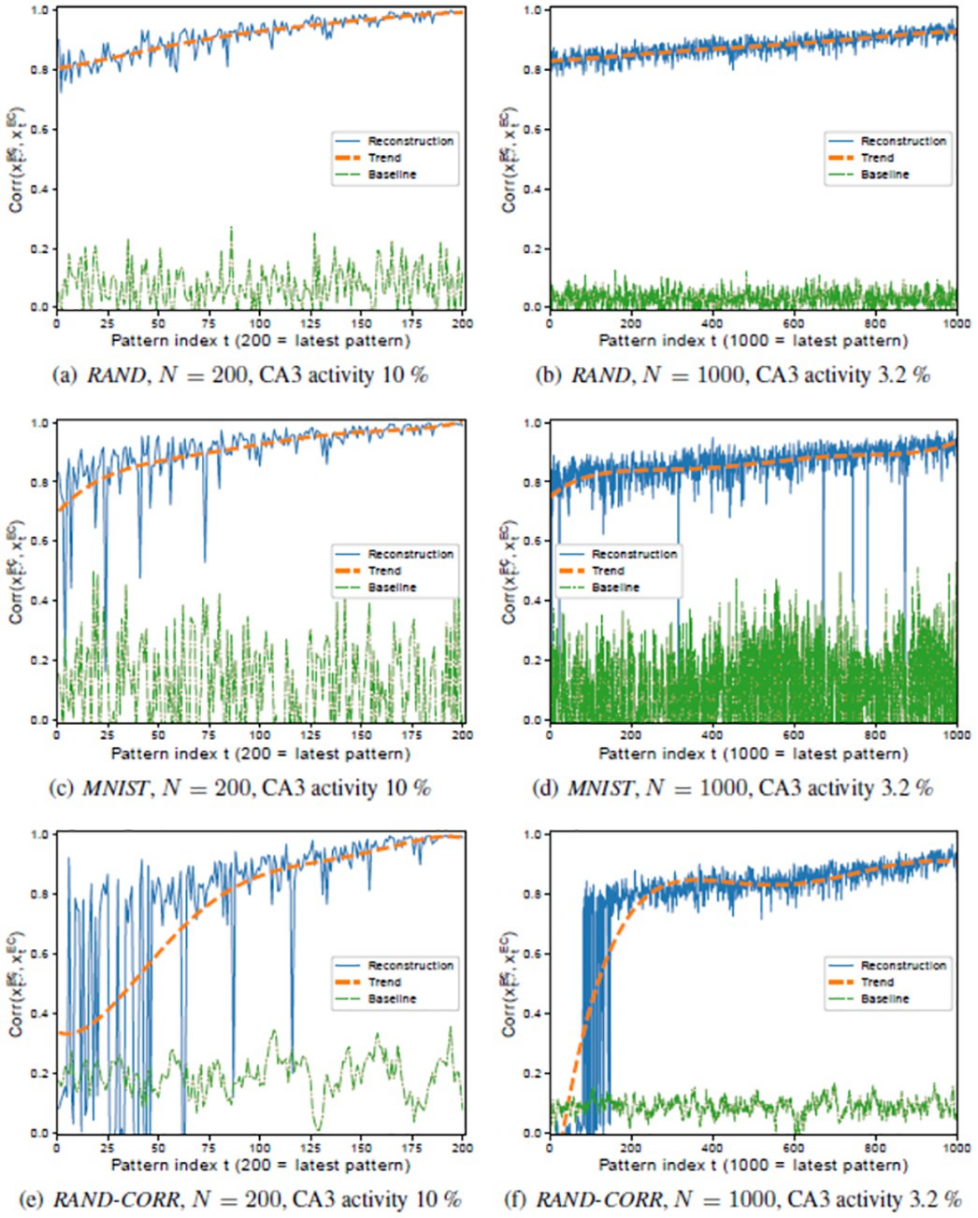
Full intrinsic performance of the HC-model (excluding subregion DG) on *RAND>* (a,b), *MNIST* (c,d), and *RAND-CORR* (e,f), with N = 200 with CA3 activity 10% (left column), and N = 1000 with CA3 activity 3.2% (right column). Apart from the CA3 activity and *N* the same setup has been used for all networks.

While the experiments shown above use an average activity of 20%, the best performance could actually be achieved with 43% activity (data not shown), but since the activity in the hippocampus (3.2%) is much lower, we have chosen 20% as the lowest activity that has almost the same performance as 43% for both network sizes. Fig 14 (right column) shows the performance when a physiological average activity of 3.2% is used for a model of size *N* = 1000, which still shows a rather good performance on all three datasets, except for early patterns in *RAND-CORR*. For *N* = 200, however, an activity of 3.2% does not suffice (data not shown), and therefore an average activity of 10% is required to reach a reasonable performance (Fig 14 left column).

This provides evidence that larger networks require less activity, so that 3.2% might actually be optimal for a network of physiological size (N = 100.000). Compared to the average activity known for the rat hippocampus, a much higher average activity level has been chosen, but at the same time also much fewer neurons per subregion. The results also confirm that the performance degrades with increasing correlations in the dataset, which is lowest for *RAND* and highest for *RAND-CORR*. If one adds noise to the patterns, the performance of the smaller networks degrades much quicker with increasing noise level than that of the larger networks (data not shown).

### 5.3 Improving the performance of the model without DG on temporally correlated data through replay

**Fig 15:**
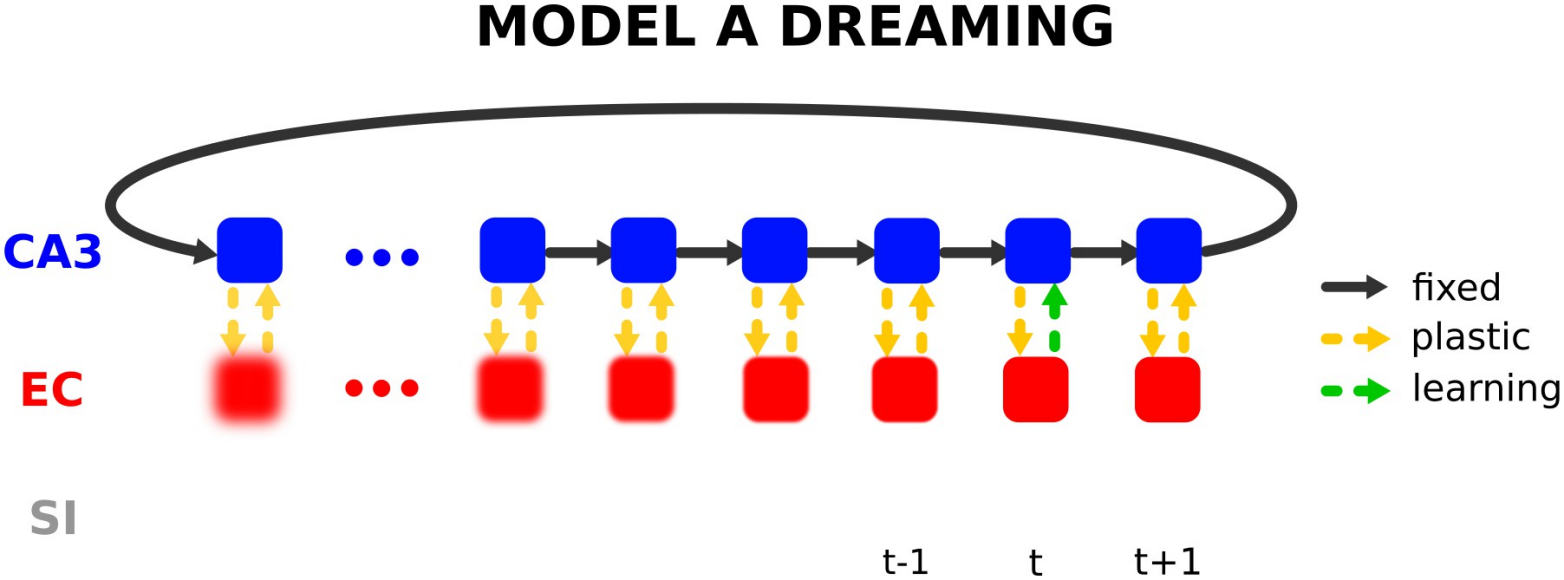
Illustration of the replay process excluding the DG subregion. The intrinsic sequence is looped through several times where at each time step *t* the EC pattern is reconstructed from the intrinsic pattern via pathway CA3 → EC and the forward pathway EC → CA3 is updated (indicated by the green dashed arrow) by hetero-associating the reconstructed EC pattern with the corresponding CA3 pattern. Through this process the quality of the EC → CA3 pathway increases leading to a better recall performance without any external input from SI.

As discussed in the previous sections, the encoder without the DG subregion cannot deal with correlated EC patterns, whereas the decoder performs equally well on uncorrelated and correlated EC patterns as the number of units in CA3 is larger than in EC. In general, the mapping from a high dimensional space to a lower dimensional space is easier than from a lower dimensional space to a higher one, in particular, it is much easier to associate from uncorrelated patterns (in CA3) to correlated patterns (in EC) than vice versa. Introducing DG helps to overcome this problem, but even without a DG subregion, the replay capability of the HC-model can be exploited here to enhance the encoding of the forward pathway EC → CA3 against the backwards path CA3 → EC such that the model can overcome the problem of correlated input stimuli. The process goes as follows: In an offline phase of the model (no external input) an intrinsic pattern **x**^*CA*3^(*t*) is selected randomly and the model recalls the corresponding EC pattern ${\tilde{\text{x}}}^{EC}\left(t\right)$. The mapping (EC → CA3) is then strengthened using hetero-associated Hebbian-descent with ${\tilde{\text{x}}}^{EC}\left(t\right)$ as input and **x**^*CA*3^(*t*) as target values (Fig 15). The intrinsic pattern triggers the next intrinsic pattern and the whole process repeats.

**Fig 16:**
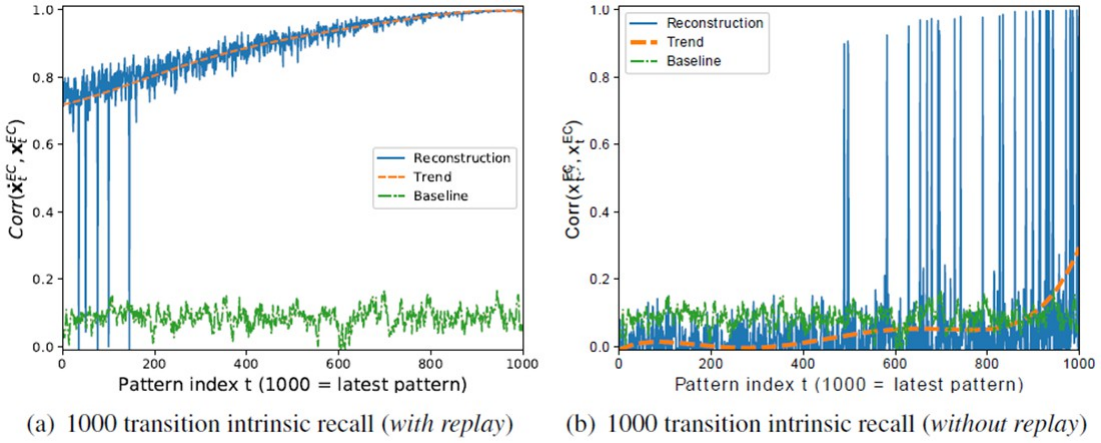
Recall performance of the simplified HC-model (excluding the DG subregion) on the *RAND-CORR* dataset (a) after retraining/replay, and (b) without replay.

For this retraining, we recall the entire sequence 10 times in sequential order, so that each pattern is seen another 10 times. Although the quality of the recalled patterns in EC is not perfect, in particular for older memories, replay helps to improve the associations stored in the EC → CA3 pathway. Even though the encoder is retrained on imperfect inputs ${\tilde{\text{x}}}^{EC}$, they seem to be good enough to improve the encoder, and the advantage of multiple training epochs seems to outweigh the disadvantage of using imperfect input patterns. This helps to improve the intrinsic recall (Fig 16). Hence, retraining the forward connections based on what is encoded in the backwards connection in an interleaved fashion improves the forward connections such that they can decorrelate the input patterns.

While the model allows us to analyze the EC → CA3 and EC → DG → CA3 pathways separately, they exist simultaneously in the hippocampus. Our results suggest that the two pathways can act as a fast online (EC → DG → CA3) and a slow offline (EC → CA3) way of hetero-associating pattern sequences, where the latter is much more efficient in terms of the number of neurons and connections. The process of replay could therefore be used to integrate all or some associations stored in pathway EC → DG → CA3 into EC → CA3. Note that replay would not be possible with Hebb’s rule or the covariance rule, as both update rules do not improve by seeing patterns several times [27].

## 6 Discussion and conclusion

To the best of our knowledge, the model presented in this study is the first neural model of the hippocampus that can continuously store and retrieve pattern sequences online in a one-shot manner while exhibiting a high capacity and a graded forgetting behavior. Notably, this model does not rely on plasticity in the recurrent connections of CA3 but rather in the feedforward connections to and from CA3 to do the association, which works better than the former case. Our findings emphasize the significance of pattern separation by DG, particularly when dealing with correlated patterns within a sequence, but this can be substituted by offline replay, even without external sensory inputs, which has the potential to enhance memory retrieval. The distinctive properties of the Hebbian Descent learning rule used in our model enable it to obtain a high storage capacity without jeopardizing it to catastrophic interference.

Several studies, inspired by biological mechanisms, have investigated the storage and retrieval of pattern sequences [45–49]. Most of these studies have primarily examined CA3 as an isolated auto-associative memory within the hippocampal circuit. They have predominantly employed random patterns to probe the memory network and, notably, none of them has considered online learning of sequences.

In contrast, our model presented in this study takes a different approach. Firstly, we use real-world datasets as input patterns, such as handwritten digits. This is significant because neural activities in real-world scenarios exhibit correlations that are characteristic of the agent’s environment. By incorporating such realistic patterns, experimental studies can be modeled in a more authentic manner, potentially leading to different conclusions [33].

Secondly, we have required the model to store pattern sequences based on only a single exposure, thereby performing one-shot learning. This is more realistic, in particular in dynamic environments [50] where pattern sequences are usually available only once but might still be relevant, for example to avoid negative experiences after a single exposure, like pain [51], even if the avoidance behavior is not necessarily an optimal one. The benefit of storing single experiences for learning a behavior has been demonstrated with functional models of episodic memory in reinforcement tasks [52, 53]. These models are not neural and simply store the incoming sequences as they are. A more neural architecture has been presented by Alabi [54], but it does not store the sequences, it only uses them to update the value function for a concrete navigation task. Our model achieves one-shot learning by hetero-associating arbitrary input stimuli with a preexisting intrinsic sequence of random patterns with the Hebbian descent learning rule. Interestingly, this learning rule automatically implies an efficient forgetting behavior.

While the model of Alabi [54] defines replay of sequences of spatial representations as a prerequisite for one-shot learning, our model with DG has the capability of one-shot learning even with the absence of replay by mapping the input pattern to the high dimensional DG space before being hetero-associated with the pattern in CA3. By this mapping, the patterns are decorrelated and any limitations in the forward connections can be compensated, rendering replay unnecessary.

Already in previous work, we have addressed the storage and recall of pattern sequences through hetero-association of input sequences with intrinsically generated sequences in CA3 [19]. However, that work focused on CA1 as an additional processing step instead of DG, and it considered offline learning rather than online learning.

The standard framework proposes that plasticity in CA3 is responsible for the storage of episodic memories in the hippocampus [14], but our results indicate that learning sequences online through associations within CA3 from one pattern to the next might be difficult to realize. A key result of our work is thus that intrinsic pattern sequences in CA3, as proposed by CRISP theory and a growing body of research [16, 54–56], is a particularly suitable basis for successful one-shot storage of pattern sequences. Intrinsic sequences resemble synfire chains, where precise spatiotemporal firing patterns composed of a sequence of synchronized firing in a series of small groups of neurons are produced, even though in cortical areas rather than in the Hippocampus [57]. In line with the common assumption that DG serves as a pattern separator, a simple generic decorrelation mechanism in our model implemented in DG through a sparsification of patterns is sufficient to overcome the problem of correlations in the input. This is consistent with the standard framework as well as the ‘context reset’ concept in the CRISP framework, which can be understood as a separation of sequences, so that the roles the two frameworks assign to DG are rather similar. However, in the CRISP framework, DG needs to initialize the intrinsic sequence in CA3 during encoding and the first pattern during retrieval, which is not the case in our model, in which the first pattern during encoding is picked randomly, and the design of the model enables retrieval from any pattern.

One key element of our model is the preexisting intrinsic sequence in CA3. This can be used to recall a stored sequence and retrain the encoder connections with the recalled patterns. This is effective in improving the encoding, because the decoding from CA3 to EC is easy to learn and already of high quality after the first association during storage. The improvement is so significant that it can even replace the decorrelation performed by DG. Replay of intrinsic sequences has been observed in hippocampus [58–60] and hypothesized to support systems consolidation of memories in cortex [61, 62], while we use it for consolidation within the hippocampus, but both could be true. Since the sequences in our model are intrinsic, they can also account for forward pre-play [63].

Decorrelation through DG and improving the direct EC-CA3 connections through replay could also work synergistically. Treves [64] have hypothesized that the input into CA3 via DG through the sparse but very strong mossy fibers could be optimal for storage while the richer but weaker perforant pathway from EC directly to CA3 might be better for retrieval. The replay mechanism presented here to improve the EC-CA3 connections could be an answer to the question of how the perforant pathway could be set up to do optimal retrieval for patterns that have been stored through the mossy fibers.

Forgetting is an important characteristic of memory in biological systems, as it minimizes information interference, hence improving the efficiency of recall [65]. While all memory networks, like the Hopfield network for example, suffer from catastrophic interference if they are overloaded and therefore require a forgetting mechanism, it still remains a question of how much forgetting is needed in the system. If there is no forgetting mechanism at all, then the system first stores everything and then breaks down at some point when it gets overloaded. If there is a lot of forgetting, then patterns get forgotten earlier than needed to free the memory. However, if there is just the right level of forgetting the system frees the memory at exactly the rate that it needs to accommodate the new incoming patterns. While weight decay is a forgetting mechanism that is often used as a remedy for catastrophic interference, parameter tuning is required in this case, and this often does not lead to the optimal solution. Our model has the advantage of optimally forgetting old patterns while maintaining a rather high capacity of approximately 43% of the number of CA3 neurons for arbitrary input data. This is due to the unique characteristics of the Hebbian Descent learning rule, where old patterns do not decay according to a fixed timescale, but it rather autonomously changes the weights only so much as the system needs to learn the new pattern as a sort of on-demand forgetting mechanism, without throwing memories away prematurely.

There are a number of aspects in the model that are not fully developed yet and should be improved: (i) We establish the robust intrinsic sequence in CA3 in a somewhat artificial way. A self-organisational model needs to be developed for this, possibly analogous to the self-organization of synfire-chains [57]. The same holds true for the EG-DG mapping. (ii) For simplicity we work with one circular intrinsic sequence. In reality, however, the hippocampus needs to store many separate sequences of various length. It is probably easy to establish a whole set of intrinsic sequences in a recurrent network, but it is unclear how a sequence of appropriate length would get selected during one-shot storage and how the system would deal with events that turn out to be longer than the selected intrinsic sequence. One could imagine that longer events are distributed over several intrinsic sequences, which are then stitched together. (iii) Real-world events unfolding in time are not well represented with a fixed frame rate, like in our and most other models.

Future work for this model is encouraged to also address autonomy, in the sense that it should exhibit a decision mechanism of when to store something and when not, have a concept of the start and end of a sequence, and at what speed the intrinsic sequence proceeds compared to an accordingly segmented input sequence; the current version of the model assumes a fixed frame rate and a one to one mapping between every frame read from the retina and an intrinsic pattern, however, a lot of models of this kind has this limitation, and one solution would be the work by Sandamirskaya [66] which addresses the temporal dynamics aspect and has a more flexible notion of temporal sequence. The input to the model is also rather a simplification and is intended for the purpose of generating reasonable stimuli that we can visually compare. (iv) Our model lacks autonomy. It does not decide by itself when to store sensory input and how long the sequences should be. (v) In this work we have assumed correct and complete patterns for storage and retrieval. It would be interesting to investigate how the system deals with degraded patterns due to representational shifts [67] or incomplete representations (e.g. [68]). Representations can be incomplete on purpose to increase memory efficiency. A complete scenario can then be recovered based on semantic information [69].

Our work presents a neural model that respects basic hippocampal anatomy (size and connectivity of subregions, locality of learning rules) and is able to perform one-shot sequence storage with high capacity and efficient forgetting behavior. It is based on three essential principles: (i) intrinsic sequences, (ii) pattern separation in DG (or a replay process instead), and (iii) Hebbian descent learning. We hope that it will contribute to a better understanding of sequential episodic memory and see potential for further development.

## Supporting information

S1 Appendix Detailed calculation flow of the hippocampus model and the simplified version of it. (PDF)
