# Correction: A neural network model for online one-shot storage of pattern sequences

**DOI:** 10.1371/journal.pone.0313130

**Published:** 2024-10-29

**Authors:** Jan Melchior, Aya Altamimi, Mehdi Bayati, Sen Cheng, Laurenz Wiskott

There are errors in the funding statement. The correct funding statement is as follows: This work was supported by grants from the German Research Foundation (DFG), project number 397530566 –FOR 2812, P5 –, project number 419037518 –FOR 2812, P2 –and project number 122679504 –SFB 874, B2 The funders had no role in study design, data collection and analysis, decision to publish, or preparation of the manuscript.

The ORCID iD for Jan Melchior is incorrect. The correct ORCID iD for Jan Melchoir is 0000-0002-6956-685X (https://orcid.org/0000-0002-6956-685X).

The ORCID iDs are missing for the second, third and fourth author. Please see the authors’ respective ORCID iDs here:

Author Aya Altamimi’s ORCID iD is: 0000-0002-7730-5940 (https://orcid.org/0000-0002-7730-5940).

Author Mehdi Bayati’s ORCID iD is: 0000-0002-4040-198X (https://orcid.org/0000-0002-4040-198X).

Author Sen Chang’s ORCID iD is 0000-0002-6719-8029 (https://orcid.org/0000-0002-6719-8029)

All authors, Jan Melchior, Aya Altamimi, Mehdi Bayati, Sen Cheng, Laurenz Wiskott, should not have been attributed equal contribution to this work.
