# Correction: Correction: A neural network model for online one-shot storage of pattern sequences

**DOI:** 10.1371/journal.pone.0318391

**Published:** 2025-01-24

**Authors:** The PLOS One Staff

## Notice of republication

This correction notice was republished on January 16, 2024, to correct an error in the references list. The publisher apologizes for the mistake. Please download this correction notice again to view the revised version. For reference, both the originally published uncorrected notice and the republished corrected notice are provided here.

## Supporting information

S1 File Originally published, uncorrected article. (PDF)

S2 File Republished, corrected article. (PDF)
