# Supplementary material for: A neural network model for online one-shot storage of pattern sequences
Source: PLoS One. 2024 Jun 20;19(6):e0304076. doi: 10.1371/journal.pone.0304076 (PMC11189254; doi:10.1371/journal.pone.0304076)
Supplement: S1 Appendix — (PDF) [file pone.0304076.s001.pdf]

S1 Appendix. Detailed calculation flow of the hippocampus model and the simplified version of it. For notational simplicity we use matrix-notation, so that the activity of all neurons within one subregion are calculated according to Eq. 1 by

$$\mathbf{h} = \boldsymbol{\phi}\left(\mathbf{W}^{T}\left(\mathbf{x} - \boldsymbol{\mu}\right) + \mathbf{b}\right). \tag{10}$$

For the hippocampus model the calculations are given by

$$\begin{aligned}
\mathbf{x}^{EC}(t) &= s\left(\mathbf{W}^{SI\to EC}\left(\mathbf{x}^{SI}(t) - \boldsymbol{\mu}^{SI}\right) + \mathbf{b}^{SI\to EC}\right), && (11)\\
\mathbf{x}^{DG}(t) &= \sigma\left(\mathbf{W}^{EC\to DG}\left(\mathbf{x}^{EC}(t) - \boldsymbol{\mu}^{EC}\right) + \mathbf{b}^{EC\to DG}\right), && (12)\\
\mathbf{x}^{CA3}(t) &= \sigma\left(\mathbf{W}^{DG\to CA3}\left(\mathbf{x}^{DG}(t) - \boldsymbol{\mu}^{DG}\right) + \mathbf{b}^{DG\to CA3}\right), && (13)\\
\mathbf{x}^{CA3}(t+1) &= \sigma\left(\mathbf{W}^{CA3\to CA3}\left(\mathbf{x}^{CA3}(t) - \boldsymbol{\mu}^{CA3}\right) + \mathbf{b}^{CA3\to CA3}\right), && (14)\\
\mathbf{x}^{EC}(t+1) &= \sigma\left(\mathbf{W}^{CA3\to EC}\left(\mathbf{x}^{CA3}(t+1) - \boldsymbol{\mu}^{CA3}\right) + \mathbf{b}^{CA3\to EC}\right), && (15)\\
\mathbf{x}^{SI}(t+1) &= \sigma\left(\mathbf{W}^{EC\to SI}\left(\mathbf{x}^{EC}(t+1) - \boldsymbol{\mu}^{EC}\right) + \mathbf{b}^{EC\to SI}\right), && (16)
\end{aligned}$$

where $s(\cdot)$ denotes the step function used to transfer the input $\mathbf{x}^{SI}(t)$ to a binary representation $\mathbf{x}^{EC}(t)$, and $\sigma(\cdot)$ denotes the logistic function ($\sigma(z) = \frac{1}{1+e^{-z}}$) used to transfer the $\mathbf{x}^{EC}(t)$ representation to the $\mathbf{x}^{CA3}(t)$ pattern. The calculation flow for the simplified model without DG for retrieving the next pattern is given by

$$\begin{aligned}
\mathbf{x}^{EC}(t) &= s\left(\mathbf{W}^{SI\to EC}\left(\mathbf{x}^{SI}(t) - \boldsymbol{\mu}^{SI}\right) + \mathbf{b}^{SI\to EC}\right), && (17)\\
\mathbf{x}^{CA3}(t) &= \sigma\left(\mathbf{W}^{EC\to CA3}\left(\mathbf{x}^{EC}(t) - \boldsymbol{\mu}^{EC}\right) + \mathbf{b}^{EC\to CA3}\right), && (18)\\
\mathbf{x}^{CA3}(t+1) &= \sigma\left(\mathbf{W}^{CA3\to CA3}\left(\mathbf{x}^{CA3}(t) - \boldsymbol{\mu}^{CA3}\right) + \mathbf{b}^{CA3\to CA3}\right), && (19)\\
\mathbf{x}^{EC}(t+1) &= \sigma\left(\mathbf{W}^{CA3\to EC}\left(\mathbf{x}^{CA3}(t+1) - \boldsymbol{\mu}^{CA3}\right) + \mathbf{b}^{CA3\to EC}\right), && (20)\\
\mathbf{x}^{SI}(t+1) &= \sigma\left(\mathbf{W}^{EC\to SI}\left(\mathbf{x}^{EC}(t+1) - \boldsymbol{\mu}^{EC}\right) + \mathbf{b}^{EC\to SI}\right), && (21)
\end{aligned}$$

Notice, that the input can also be a retrieved pattern or a corrupted input denoted by $\dot{\mathbf{x}}$ and $\tilde{\mathbf{x}}$, respectively. Of course during sequence recall, Eqs. 14 and 19 are iterated, before moving on to Eqs. 15 and 16, and Eqs. 20 and 21, respectively.

S1 Table. Model and parameters description

Learning rule	Hebbian Descent.
Network connectivity	A fully connected 3 layer auto-encoder network with tied weights.
Neuron model	Rate-based model (each neuron is represented by a centered artificial neuron).
a_j	Membrane potential.
b_j	Bias.
c_i	Hidden bias.
h_j	Output activity.
t_j	Target output activity.
w_{ij}	Weight.
x_i	Input activity.
η	Learning rate.
λ_j	Hidden offset (i.e. the mean of the output unit j).
$\tilde{\lambda}_j$	Target average hidden activity.
μ_i	Offset value (usually the target activity of the input layer approximating the mean).
$\phi(\cdot)$	Activation function (a sigmoid function).
